# A novel rapamycin analog is highly selective for mTORC1 in vivo

**DOI:** 10.1038/s41467-019-11174-0

**Published:** 2019-07-19

**Authors:** Katherine H. Schreiber, Sebastian I. Arriola Apelo, Deyang Yu, Jacqueline A. Brinkman, Michael C. Velarde, Faizan A. Syed, Chen-Yu Liao, Emma L. Baar, Kathryn A. Carbajal, Dawn S. Sherman, Denise Ortiz, Regina Brunauer, Shany E. Yang, Stelios T. Tzannis, Brian K. Kennedy, Dudley W. Lamming

**Affiliations:** 1 Buck Institute for Research on Aging, Novato, CA 94945 USA; 2 Department of Dairy Science, University of Wisconsin-Madison, Madison, WI 53706 USA; 3 Department of Medicine, University of Wisconsin-Madison, Madison, WI 53705 USA; 4 William S. Middleton Memorial Veterans Hospital, Madison, WI 53705 USA; 5 Molecular and Environmental Toxicology Program, University of Wisconsin-Madison, Madison, WI 53706 USA; 6 Aeonian Pharmaceuticals, Inc., San Francisco, CA 94598 USA; 7 Masters of Science in Biology Program, Dominican University, San Rafael, CA 94901 USA; 8 University of Wisconsin Carbone Cancer Center, Madison, WI 53705 USA; 9 Present Address: Institute of Biology, University of the Philippines, Quezon City, NCR Philippines; 10 Present Address: Texas A&M University, College Station, TX 77843 USA

**Keywords:** Drug discovery, Metabolism

## Abstract

Rapamycin, an inhibitor of mechanistic Target Of Rapamycin Complex 1 (mTORC1), extends lifespan and shows strong potential for the treatment of age-related diseases. However, rapamycin exerts metabolic and immunological side effects mediated by off-target inhibition of a second mTOR-containing complex, mTOR complex 2. Here, we report the identification of DL001, a FKBP12-dependent rapamycin analog 40x more selective for mTORC1 than rapamycin. DL001 inhibits mTORC1 in cell culture lines and in vivo in C57BL/6J mice, in which DL001 inhibits mTORC1 signaling without impairing glucose homeostasis and with substantially reduced or no side effects on lipid metabolism and the immune system. In cells, DL001 efficiently represses elevated mTORC1 activity and restores normal gene expression to cells lacking a functional tuberous sclerosis complex. Our results demonstrate that highly selective pharmacological inhibition of mTORC1 can be achieved in vivo, and that selective inhibition of mTORC1 significantly reduces the side effects associated with conventional rapalogs.

## Abstract (web summary)

Rapamycin extends lifespan in model organisms by targeting mTORC1, but exerts off-target side effects via inhibition of mTORC2. Here, the authors report the identification of a selective mTORC1 inhibitor, and show that it inhibits mTORC1 activity both in vitro and in vivo, with reduced side effects on glucose homeostasis, lipid metabolism, and the immune system.

## Introduction

Age-related diseases, including cardiovascular disease, cancer, neurodegenerative disease, and type 2 diabetes, represent the greatest healthcare challenges in a graying world. Targeting the aging process through dietary or pharmacological interventions has been advanced as way to prevent or delay many age-related diseases simultaneously, extending healthspan. Over the past decade, significant scientific activity has focused on the anti-aging potential of rapamycin, an inhibitor of the serine/threonine protein kinase mTOR (mechanistic Target Of Rapamycin), which can extend the lifespan of organisms including yeast, worms, flies, and mice^1–4^. Excitingly, rapamycin also prevents or delays the onset of age-related diseases in mice, including cancer and Alzheimer’s disease, rejuvenating the aging mouse heart, and ameliorating age-related cognitive decline^5–9^. Scientists are now beginning to explore the possibility that rapamycin may be of therapeutic benefit for age-related diseases in dogs and humans^10–12^.

Unfortunately, treatment with rapamycin and its analogs (rapalogs) is associated with negative side effects that limit their potential utility as anti-aging therapies. These side effects include immunosuppression, glucose intolerance, an increased risk of type 2 diabetes, and disruption of lipid homeostasis^13–19^. Although short term, low dose, and/or intermittent rapalog-based regimens may partially limit side effects^11,20–22^, and trials of rapamycin in the elderly have begun^11,22,23^, the efficacy of such treatment regimens in treating age-related diseases remains to be determined, particularly as the effects of rapamycin on longevity are dose-dependent^24,25^. All three FDA-approved rapalogs—sirolimus, everolimus, and temsirolimus—have similar effects on the glucose metabolism and immune cell profile of mice^20^. It is clear that there is an urgent need for new molecules that inhibit mTOR signaling with reduced side effects^26^.

mTOR is found in two distinct protein complexes. mTORC1, which is acutely sensitive to rapamycin, promotes cell growth, ribosomal biogenesis, and protein translation through substrates that include S6K1 and 4E-BP1, and regulates autophagy via ULK1. mTORC2, originally characterized as rapamycin resistant^27^, functions primarily as a downstream effector of the insulin/IGF-1 signaling pathway, through substrates that include AKT^15^. Several years ago, while investigating the molecular basis of rapamycin-mediated glucose intolerance, we made the surprising discovery that long-term treatment with rapamycin inhibits not only mTORC1 but also inhibits mTORC2 in vivo in many metabolically active tissues^28^. This discovery, in combination with extensive results from genetic manipulation of model organisms, has led to the consensus that the beneficial effects of rapamycin on health and longevity are mediated solely by inhibition of mTORC1, while the off-target inhibition of mTORC2 is responsible for many of the negative side effects of rapamycin. Thus, many of the side effects of rapalogs could be avoided by selectively inhibiting mTORC1 signaling^14^.

Here, we report the identification of DL001, a rapamycin analog with much greater selectivity for mTORC1 than rapamycin. We show that DL001 selectively inhibits mTORC1 both in cell culture and in vivo in mice. Critically, we find that unlike rapamycin, DL001 does not disrupt glucose or lipid homeostasis, and has substantially reduced effects on the immune system. Our results demonstrate that highly selective pharmacological inhibition of mTORC1 can be achieved in vivo, and that such a compound minimizes the negative metabolic and immunological impacts associated with rapamycin and other conventional rapalogs.

## Results

### Identification and characterization of DL001 in vitro

**Fig. 1:**
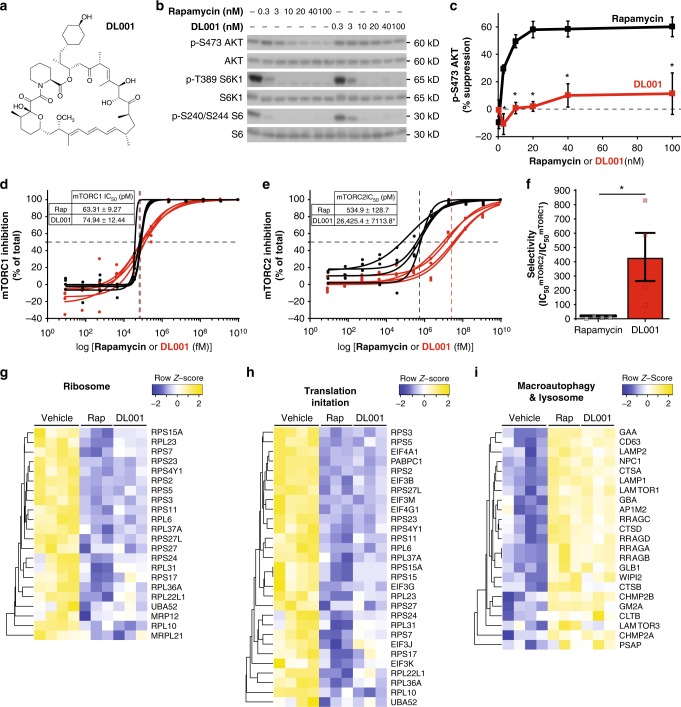
DL001 is a selective inhibitor of mTORC1. **a** Structure of DL001. **b**, **c** PC3 cells were treated with rapamycin or DL001 at 0.3–100 nM for 48 h and **b** the phosphorylation of S6K1 T389 (a mTORC1 substrate), S6 S240/S244 (a readout for mTORC1 activity), and AKT S473 (a mTORC2 substrate) was determined by western blotting; **c** the inhibition of AKT S473 phosphorylation was plotted (*n* = 3 biologically independent experiments; *p < 0.005, Sidak’s test following two-way ANOVA). Rapamycin data are plotted in black, DL001 data plotted in red. **d**–**f** The IC_50_ for rapamycin (Rap) and DL001 against **d** mTORC1 and **e** mTORC2 was determined using an in vitro assay to measure the phosphorylation of S6 S240/S244 (mTORC1) and AKT S473 (mTORC2) in PC3 lysates treated for 24 h. Rapamycin data are plotted in black, DL001 data plotted in red. **f** The selectivity of each compound for mTORC1 was calculated from the IC_50_ (*n* = 4 biologically independent replicates, three technical replicates each; IC_50_ was calculated using Prism 7; *p < 0.05, Student’s *t*-test). **g**–**i** Quantitative proteomic analysis was performed on PC3 cells treated for 24 h with 100 nM Rapamycin (Rap) or DL001, or vehicle, and significantly affected (*q* < 0.05) proteins were analyzed with gProfiler to identify significantly altered KEGG and Reactome categories, which included **g** ribosome, **h** translation initiation, and **i** macroautophagy and lysosome. Error bars represent standard error. Source data are provided as a Source Data file

Although all FDA-approved rapalogs inhibit both mTOR complexes^20,29^, recent work investigating the molecular mechanism by which rapamycin disrupts mTORC2 (ref. ^30^) suggested that rapamycin derivatives with reduced activity against mTORC2 but full potency against mTORC1 might exist. We therefore screened a library of approximately 90 rapamycin analogs in order to identify compounds with reduced activity against mTORC2; for the screens, we primarily utilized PC3 cells (Supplementary Fig. 1a), a cell line in which mTORC2 is exquisitely sensitive to rapamycin^31^. While a majority of the compounds demonstrated strong inhibition of mTORC1 (as indicated by reduced phosphorylation of the mTORC1 downstream readout S6 S240/S244), we also identified several compounds with reduced inhibition of mTORC2. We selected DL001 (Fig. 1a) for further characterization due to its strong mTORC1 inhibitory activity.

We compared the effect of rapamycin and DL001 on mTORC1 and mTORC2 activity in PC3 cells across a range of doses (0.3–100 nM) via western blotting, assessing the phosphorylation of the mTORC1 substrate S6K1 and its substrate S6, the phosphorylation of rapamycin-sensitive and -insensitive residues on 4E-BP1 (S65 and T37/S46, respectively), and the phosphorylation of the mTORC2 substrate AKT S473. While rapamycin and DL001 had very similar effects on mTORC1 activity (Fig. 1b, Supplementary Fig. 1b–d), the effect of these compounds on mTORC2 was dramatically different (Fig. 1b, c). The IC_50_^mTORC2^ for rapamycin in this assay was approximately 10 nM, while the IC_50_^mTORC2^ for DL001 was unable to be determined, as the experiments did not achieve greater than 50% inhibition at the levels tested, but was in excess of 100nM. We observed similar effects on other mouse and human cell lines (Supplementary Fig. 2a, b).

In order to validate the above western blot analysis and to determine accurately the IC_50_^mTORC2^ for DL001, we utilized a commercially available high-sensitivity chemiluminescent assay (AlphaLISA). PC3 cells treated with either vehicle or a range of doses of either rapamycin or DL001 for 24 h were analyzed to determine the phosphorylation of S6 S240/S244 and Akt S473. As shown in Fig. 1d, in our assay we found that rapamycin inhibited mTORC1 with an IC_50_ of 63.3 pM, whereas DL001 inhibited mTORC1 with a very similar IC_50_ of 74.9 pM. In contrast, while rapamycin inhibited mTORC2 activity with an IC_50_ of 534.9 pM, DL001 inhibited with a significantly greater IC_50_ of 26,245.4 pM (Fig. 1e). Thus, while both rapamycin and DL001 are similarly potent mTORC1 inhibitors, DL001 is over 430× more selective for mTORC1 than mTORC2—and is 44-fold more selective for mTORC1 than rapamycin (Fig. 1f).

Finally, to comprehensively compare the functional impact of rapamycin and DL001 treatment on cellular processes downstream of mTORC1, we performed quantitative proteomics. We observed that rapamycin and DL001 both significantly downregulated ribosomal proteins and proteins involved in translation (Fig. 1g, h**)**, while upregulating proteins involved in macroautophagy and lysosomal function (Fig. 1i).

**Fig. 2:**
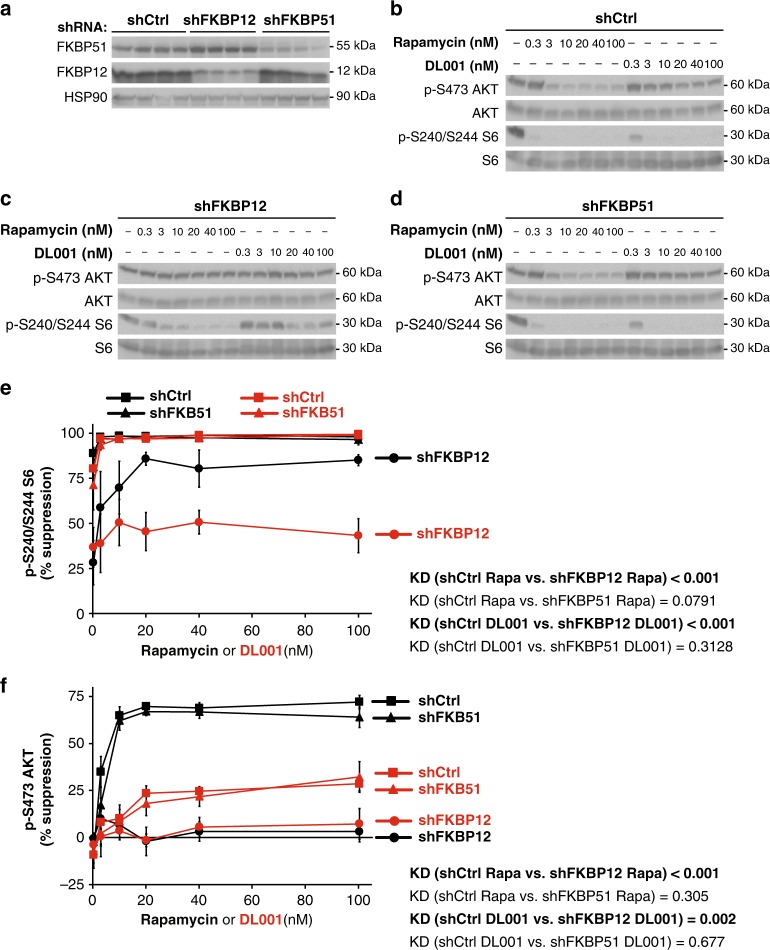
DL001 is an FKBP12-dependent mTORC1 inhibitor. **a** Decreased protein expression of FKBP12 and FKBP51 by shRNA was verified via western blotting. **b**–**d** PC3 cells expressing **b** a nonspecific control shRNA (shCtrl), **c** shRNA against *FKBP12*, or **d** shRNA against *FKBP51* were treated with rapamycin or DL001 at 0.3–100 nM for 48 h and the phosphorylation of S6 S240/S244 (a readout for mTORC1 activity) and AKT S473 (a mTORC2 substrate) was determined by western blotting. **e**–**f** The inhibition of **e** S6 S240/S244 phosphorylation and **f** The inhibition of AKT S473 phosphorylation was plotted (*n* = 3 biologically independent experiments; statistics for the overall effects of knockdown of FKBP12 or FKBP51 represent the *p*-value from a two-way ANOVA). Rapamycin data are plotted in black, DL001 data plotted in red; squares represent cells expressing shCtrl, circles cells expressing shFKBP12, and triangles cells expressing shFKBP51. Error bars represent standard error. Source data are provided as a Source Data file

Rapamycin analogs act acutely to inhibit mTORC1 via a noncompetitive mechanism that involves the formation of a ternary complex between a FK506-binding protein (FKBP), rapamycin, and mTOR^32^. While rapamycin’s activity is most closely associated with FKBP12, it was recently shown that other FKBPs, including FKBP51, can also be induced by rapamycin to bind to and inhibit mTOR activity^33^. These findings, and the recent demonstration that FKBP12 is essential for the inhibition of mTORC2 by rapamycin^30^, have led some of us to suggest that rapamycin derivatives that do not bind to FKBP12 would be more selective for mTORC1. We tested if DL001 might act independently of FKBP12 by treating PC3 cells expressing shRNAs against either FKBP12, FKBP51, or a nonspecific control with a range of doses (0.3–100 nM) of DL001 and rapamycin, and western blotting to determine the phosphorylation of S6 and AKT S473 (Fig. 2a–d). We find that knockdown of FKBP12, but not FKBP51, inhibits the ability of both rapamycin and DL001 to inhibit the phosphorylation of S6 (Fig. 2e). Likewise, the effect of both compounds on mTORC2 was completely dependent upon FKBP12, and was unaffected by knockdown on FKBP51 (Fig. 2f).

### DL001 specifically inhibits mTORC1 in vivo

**Fig. 3:**
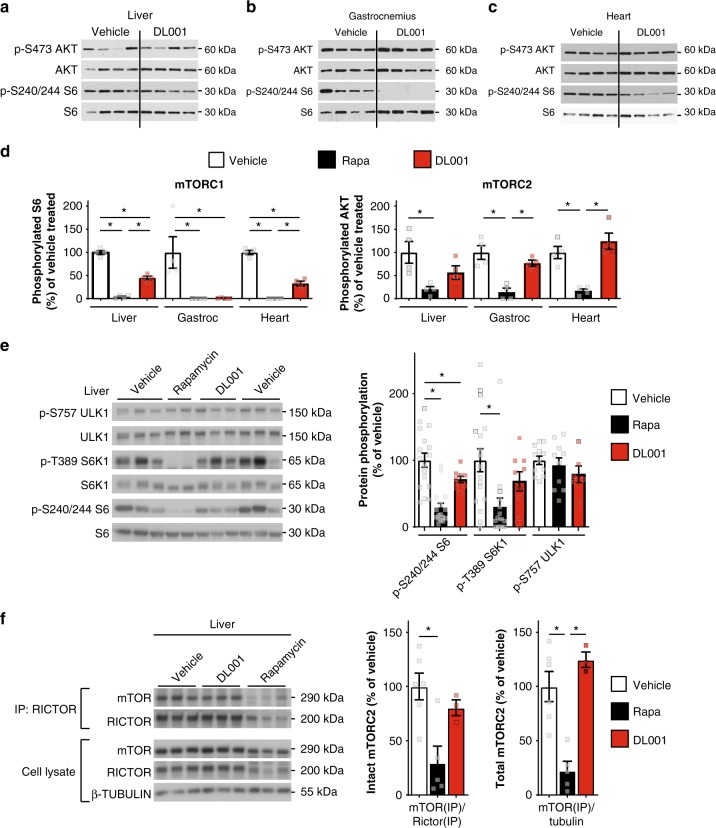
DL001 selectively inhibits mTORC1 in vivo. **a**–**d** Protein lysates were prepared from the **a** liver, **b** gastrocnemius, and **c** heart of female C57BL/6J mice treated with vehicle or 13 mg kg^−1^ DL001 every other day for 20 days, and the phosphorylation of S240/S244 S6 (a readout for mTORC1 activity) and Akt S473, an mTORC2 substrate, was determined by western blotting. **d** The activity of DL001 and rapamycin against mTORC1 and mTORC2 was determined by quantification of the western blots above and from mice treated in parallel with 8 mg kg^−1^ rapamycin ^30^ using NIH ImageJ (*n* = 4 biologically independent animals per group, *p < 0.05, Tukey–Kramer test following one-way ANOVA). Data from Vehicle-treated mice are plotted with white bars, rapamycin-treated mice with black bars, and DL001-treated mice with red bars. **e**, **f** C57BL/6J mice were treated with either vehicle, 8 mg kg^−1^ rapamycin or 12 mg kg^−1^ DL001 every other day for 5 weeks. **e** Quantification of phosphorylated liver proteins in vehicle, rapamycin, and DL001-treated mice (*n* = 17 vehicle, 16 rapamycin, and 9 DL001-treated biologically independent animals for quantification of phosphorylated S6 and S6K1, 12 vehicle, 10 rapamycin, and 6 DL0001-treated biologically independent animals for quantification of phosphorylated ULK1; Dunnett’s test following one-way ANOVA, *p < 0.05). **f** The integrity of mTORC2 was determined by immunoprecipitation of Rictor from liver lysate; the immunoprecipitate and lysate were probed with antibodies against the indicated proteins and quantified with NIH ImageJ. mTOR(IP) and RICTOR(IP) refer to the quantification of the mTOR and RICTOR immunoblots from the RICTOR immunoprecipitate (*n* = 6 vehicle, 5 rapamycin, and 3 DL001-treated biologically independent animals, *p < 0.05, Tukey–Kramer test following one-way ANOVA). **e**, **f** Data from Vehicle-treated mice are plotted with white bars, rapamycin-treated mice with black bars, and DL001-treated mice with red bars. Error bars represent standard error. Source data are provided as a Source Data file

We and others have observed that the metabolic effects of rapamycin are apparent in mice after 2–3 weeks of chronic treatment^28,34^. We therefore administered DL001 to mice for 20 days, and broadly assessed the effect of DL001 on mTORC1 and mTORC2 signaling in 11 tissues. We observed robust inhibition of mTORC1 signaling by DL001 in 9 of 11 tissues, including liver, gastrocnemius, and heart (Fig. 3a–d), visceral white adipose tissue, pancreas, soleus, lung, thymus, and kidney (Supplementary Fig. 3a–f). DL001 did not inhibit mTORC1 signaling in either stomach or spleen (Supplementary Fig. 3g–h), tissues in which rapamycin also has limited or no efficacy^30^. As compared to mice treated in parallel with 8 mg kg^−1^ rapamycin^30^, DL001 inhibited mTORC1 with equal efficacy to rapamycin in many tissues including muscle and white adipose tissue, but less strongly in liver and heart (Fig. 3d). Strikingly, mTORC2 inhibition by DL001 in every tissue was significantly reduced relative to rapamycin. Further, in tissues where complete mTORC1 inhibition by DL001 was observed (e.g. visceral fat and thymus (Supplementary Fig. 3a, e)), AKT S473 phosphorylation was increased, consistent with expectations for a highly selective mTORC1 inhibitor due to the mTORC1-mediated feedback regulation of IRS1 (refs. ^35,36^).

As the effect of rapamycin on mTORC1 and mTORC2 signaling in the liver is likely to be highly important—the liver is a key tissue in the regulation of both glucose and lipid homeostasis—we examined in detail the effect of 12 mg kg^−1^ DL001 administered every other day for 5 weeks on hepatic mTORC1 signaling. Consistent with our findings above, we confirmed decreased mTORC1 signaling in the liver of both rapamycin and DL001-treated mice as measured via the phosphorylation of S6 S240/S244 (Fig. 3e), and also observed decreased phosphorylation of the direct rapamycin-sensitive mTORC1 substrate S6K1 T389; phosphorylation of the rapamycin-resistant mTORC1 substrate ULK1 S757 trended lower in DL001-treated mice, but was not significantly decreased by either compound (Fig. 3e). Despite dosing DL001-treated mice with higher levels of compound than rapamycin-treated mice, we observed that the phosphorylation of mTORC1 substrates in a number of tissues were lower in rapamycin-treated than in DL001-treated mice; consistent with this, and preliminary observations of a shorter half-life for DL001 than rapamycin, we observed lower levels of DL001 than rapamycin in the blood 16 h following the final administration of the compounds (Supplementary Fig. 4a).

In order to directly determine the effects of chronic rapamycin treatment on mTORC2, we immunoprecipitated the complex in order to observe the association of mTOR and Rictor, the two defining subunits of mTORC2 (refs. ^28,30^). Rapamycin dramatically impacted both the amount of Rictor-associated mTOR and the total amount of mTORC2 in the liver, while DL001 had no effect on mTORC2 integrity or abundance (Fig. 3f). Genetic experiments from a number of labs have also pointed to an important role for mTORC2 in several tissues, including skeletal muscle and white adipose tissue, in the regulation of glucose homeostasis^37–40^. In agreement with our previous observation that rapamycin disrupts the Rictor–mTOR association in skeletal muscle and white adipose tissue, we observed that rapamycin, but not DL001, disrupts mTORC2 integrity in these two tissues (Supplementary Fig. 5a, b). DL001 thus shows enhanced specificity for mTORC1 not only in cell culture, but also in vivo.

### DL001 has reduced side effects relative to rapamycin

Our initial hypothesis was that a rapalog that specifically inhibits mTORC1, such as DL001, would enable us to avoid many of the side effects of rapamycin. To test this, we examined the effects of DL001 on glucose metabolism, lipid metabolism, and the immune system. Male C57BL/6J mice, which show a robust metabolic response to rapamycin and to genetic inhibition of hepatic mTORC2 signaling^28,41^, were treated for 5 weeks with either 8 mg kg^−1^ rapamycin or 12 mg kg^−1^ DL001. The specific doses of compounds used were chosen as a preliminary single dose pharmacokinetic study suggested that DL001, when delivered i.p. at 12 mg kg^−1^, has a shorter half-life in blood than rapamycin, with a *t*_1/2_ of ~3.5 h. As we expected, while there was not a significant difference between blood levels of DL001 and rapamycin 16 h after administration, blood levels of DL001 trended ~25% lower (Supplementary Fig. 4a). Given our half-life estimate, this time point reflects a near-trough concentration (*C*_min_) of DL001; the trough level of rapalogs is clinically monitored in patients subjected to rapalog therapy and is directly linked with rapalog exposure, efficacy, and side effects^42,43^. Importantly, the blood levels of both DL001 and rapamycin were substantially higher than levels observed in the blood of mice fed 14 ppm rapamycin^24^, which as we have previously demonstrated is sufficient to inhibit mTORC2 in vivo^34,44^.

**Fig. 4:**
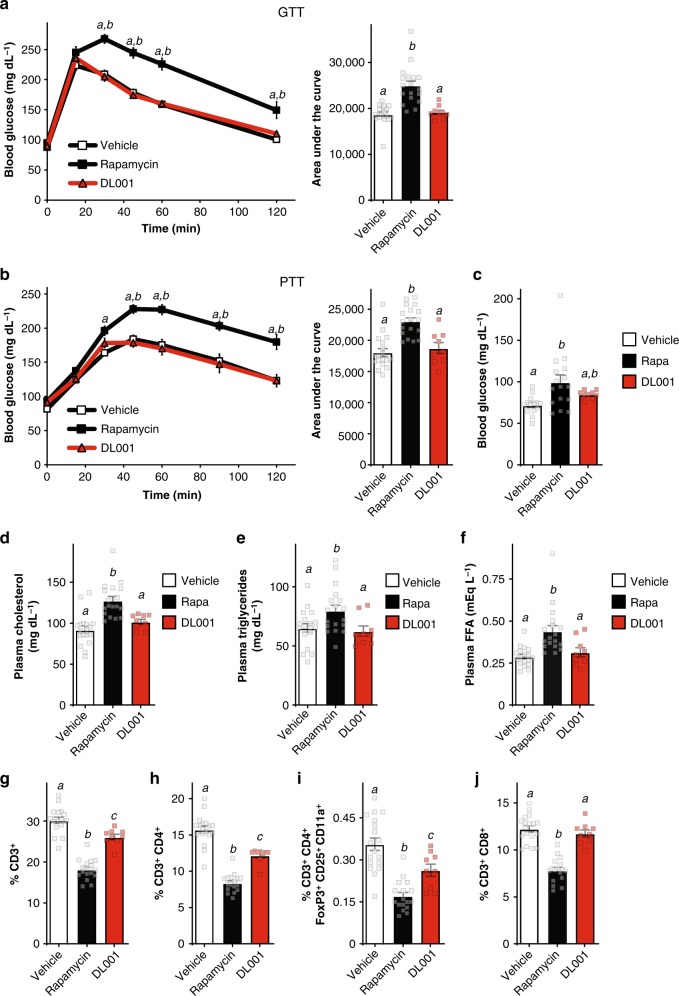
Unlike rapamycin, DL001 does not cause metabolic disruption. **a–i** C57BL/6J mice were treated with vehicle, 8 mg kg^−1^ rapamycin, or 12 mg kg^−1^ DL001 every other day. **a** Glucose and **b** pyruvate tolerance tests were performed after 2 or 3 weeks, respectively (*n* = 18 vehicle, 18 rapamycin, and 9 DL001-treated biologically independent animals; for GTT/PTT, Tukey–Kramer test following two-way repeated-measures ANOVA, a = *p* < 0.006 vs. vehicle, b = *p* < 0.006 vs. DL001. For AUC, means with the same letter are not significantly different from each other (Tukey–Kramer test following one-way ANOVA, *p* < 0.0007)). For GTT/PTT, data from Vehicle-treated mice are plotted with white squares, Rapamycin-treated mice with black squares, and DL001-treated mice with red triangles. **c** Fasting blood glucose was measured in mice after 4 weeks of treatment (*n* = 14 vehicle, 16 rapamycin, and 9 DL001-treated biologically independent animals, means with the same letter are not significantly different from each other, Tukey–Kramer test following one-way ANOVA, *p* < 0.05). **d**–**f** Blood was collected after 5 weeks of treatment and plasma levels of **d** cholesterol, **e** triglycerides, and **f** free fatty acids were determined (*n* = 18 vehicle, 18 rapamycin, and 9 DL001-treated biologically independent animals, means with the same letter are not significantly different from each other, Tukey–Kramer test following one-way ANOVA, *p* < 0.05). **g**–**i** Flow cytometry analysis (expressed as percent of total live cells) of splenocytes collected and isolated after 5 weeks of treatment (*n* = 18 vehicle, 17 rapamycin, and 9 DL001-treated biologically independent animals, means with the same letter are not significantly different from each other, Tukey–Kramer test following one-way ANOVA, *p* < 0.05). **a**–**i** Data from Vehicle-treated mice are plotted with white bars, rapamycin-treated mice with black bars, and DL001-treated mice with red bars. Error bars represent standard error. Source data are provided as a Source Data file

After 2 weeks of treatment, a fasting glucose tolerance test (GTT) was conducted (Fig. 4a); as expected, rapamycin-treated mice had significantly impaired glucose tolerance, with increased blood glucose levels starting at 30 min, and a 33% increase in total glucose burden over the time course of the assay as measured by area under the curve (AUC). In contrast, mice treated with DL001 were indistinguishable from vehicle-treated mice at every time point and in AUC (Fig. 4a). We also performed a pyruvate tolerance test (PTT); as we have previously observed^28,44^, rapamycin-treated mice were pyruvate intolerant, indicating a failure to suppress hepatic gluconeogenesis (Fig. 4b). Once again, DL001-treated mice were similar to vehicle-treated mice throughout the assay, and exhibited an AUC that was indistinguishable from that of vehicle-treated control mice (Fig. 4b). Clinically, the first sign of impaired glycemic control in humans is fasting hyperglycemia, which is induced by rapamycin in both humans and mice;^14^ we observed a statistically significant elevation in fasting glucose levels in rapamycin-treated mice, but not in mice treated with DL001 (Fig. 4c).

A prominent clinical effect of rapamycin is dyslipidemia, as defined by elevated levels of cholesterol, triglycerides, and free fatty acids; some of these effects are associated with inhibition of mTORC2 signaling, while the molecular basis of others is unclear^45^. We observed that while rapamycin robustly elevated plasma cholesterol, triglycerides, and free fatty acids, DL001 does not increase blood levels of these lipids (Fig. 4d–f). The physiological and molecular mechanisms which mediate rapamycin-induced hyperlipidemia and hypercholesterolemia have remained mysterious for some time^46^. It has been suggested that these changes may be due in part to the induction of lipolysis in white adipose tissue^45^; mTORC1 is an important regulator of lipolysis in adipocytes^47,48^, and some researchers have also proposed a role for mTORC2 in the regulation of lipolysis in adipose tissue^37,40^. Although DL001 does not inhibit mTORC2 in white adipose tissue (Supplementary Figs. 3a and 4a), rapamycin induces a 24% increase in ATGL protein, with a 16% increase (*p* = 0.1, *t*-test) in DL001-treated animals; rapamycin and DL001 both increase the phosphorylation of PKA substrates (Supplementary Fig. 6a, b). Our observations support the conclusion that mTORC1 regulates lipolysis in adipose tissue, but suggest that this effect is not sufficient to cause rapamycin-induced dyslipidemia.

In addition to its metabolic side effects, the immunosuppressive effects of rapamycin—mediated in part via inhibition of mTORC2 (ref. ^49^)—are likely a significant barrier to its application for the treatment of chronic age-related diseases. Rapamycin is associated with an increase in viral and fungal infections in humans, short courses of low-dose rapamycin impairs the defense against acute bacterial and viral infections in mice, and chronic treatment with rapamycin impairs adaptive immunity in mice^50–52^. As both mTOR complexes play important roles in adaptive immunity, in part by promoting the survival, differentiation, activation, and function of T cells^49^, we predicted that by more selectively targeting mTORC1, DL001 would have reduced—although not zero—effects on immune cell numbers as compared to rapamycin.

To compare the effects of rapamycin and DL001 on immune cells, we isolated splenocytes during tissue harvesting, and analyzed their population by flow cytometry^20^. As we and others have previously reported^20,53^, rapamycin-treated mice had significantly decreased T cell numbers; rapamycin reduced total CD3^+^ (T) cell numbers by 40%, and CD3^+^CD4^+^ (helper T) cell numbers by 47% (Fig. 4g, h). In contrast, DL001 had a significantly smaller effect on total T cell and helper T cell numbers, reducing the number of CD3^+^ cells and CD3^+^CD4^+^ cells by 13% and 23%, respectively. We and others have previously reported that chronic rapamycin treatment of mice results in decreased numbers of CD3^+^CD4^+^CD25^+^Foxp3^+^ T regulatory cells (Tregs)^20,53,54^; rapamycin reduced Tregs by 54%, while DL001 had a significantly smaller effect on Tregs, reducing the numbers by 37% (Fig. 4i). Finally, and surprisingly, while rapamycin reduced the CD3^+^CD8^+^ (suppressor/cytotoxic T) cell number by 36%, DL0001 had no effect on CD3^+^CD8^+^ cell number (Fig. 4j). While further work is required to fully assess the effect of DL001 on immune function, these observations support the idea that mTORC1-specific inhibitors such as DL001 are likely to have reduced impact on the immune system.

### DL001 suppresses the hyperactive mTORC1 of cells lacking TSC

While mTORC1-specific inhibitors such as DL001 are likely to prove of use for many diseases of aging, an immediate urgent need where they may prove beneficial is for the treatment of tuberous sclerosis complex (TSC), a rare genetic disease resulting from a loss of function mutation in *TSC1* or *TSC2* characterized by the formation of non-malignant tumors in organs including the brain, heart, kidney, and lungs; disfiguring facial angiofibromas; and neurological symptoms including seizures and epilepsy. Functioning together, the proteins encoded by *TSC1* and *TSC2* normally act to inhibit the activity of mTORC1; TSC patients therefore have hyperactive mTORC1 signaling^55^.

Treatment with rapamycin can suppress many of the effects of TSC in animal models of the disease^56^, and a rapalog (everolimus) has been FDA approved for the treatment of specific symptoms of TSC^57,58^. However, suppression of TSC symptoms requires continuous chronic treatment with rapalogs, which can results in significant side effects. Over the course of 4 years, 30% of subjects in one trial of rapalogs for TSC developed hypercholesterolemia;^59^ in a second study 5 of 18 (27%) participants were hospitalized for pneumonia over the course of 4 years^60^. One clinical trial of a rapalog for TSC had a particularly high incidence of side effects, with 72% of the subjects developing hypercholesterolemia, 66% developing hyperlipidemia, and 22% of subjects developing hyperglycemia; in that trial one subject died as a result of bacterial sepsis^13^. Thus, TSC represents a disease in which minimizing the side effect of rapamycin may be particularly important.

**Fig. 5:**
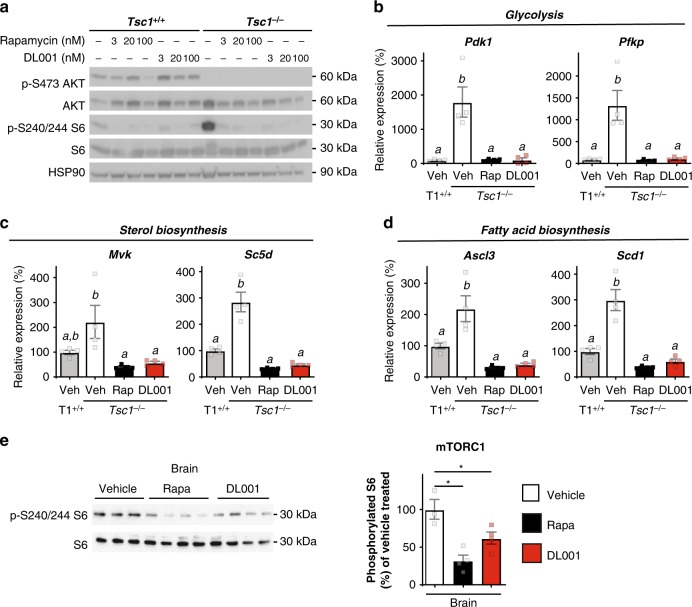
DL001 corrects mTORC1-mediated signaling and metabolic defects of *TSC1*^*−/−*^ cells. **a** Immortalized mouse embryonic fibroblasts (MEFs) expressing or lacking *TSC1* were treated with varying doses of rapamycin or DL001 for 24 h. The phosphorylation of S240/S244 S6 (a readout for mTORC1 activity) and Akt S473, an mTORC2 substrate, was determined by western blotting of protein lysate. **b**–**d** MEFs expressing (T1^+/+^) or lacking *TSC1* (*TSC1*^*−/−*^) were treated with vehicle, 100 nM rapamycin, or 100 nM DL001 for 24 h, and the expression of mTORC1 responsive genes involved in **b** glycolysis, **c** sterol biosynthesis, and **d** fatty acid biosynthesis were determined by qPCR (*n* = 4 biologically independent experiments per condition, means with the same letter are not significantly different from each other, Tukey–Kramer test following one-way ANOVA, *p* < 0.05). **e** Protein lysates were prepared from the brain of C57BL/6J mice treated with either vehicle, 8 mg kg^−1^ rapamycin or 12 mg kg^−1^ DL001 every other day for 5 weeks, and the phosphorylation of S240/S244 S6 was determined by western blotting (*n* = 3 vehicle, 4 rapamycin, and 4 DL001-treated biologically independent animals, Tukey–Kramer test following one-way ANOVA, *p < 0.05). Data from Vehicle-treated mice are plotted with white bars, rapamycin-treated mice with black bars, and DL001-treated mice with red bars. Error bars represent standard error. Source data are provided as a Source Data file

As a proof of principle, we compared the effect of rapamycin and DL001 on mouse embryonic fibroblasts (MEFs) lacking *Tsc1*. We observed that both rapamycin and DL001 effectively suppressed the hyperactive mTORC1 activity of *Tsc1*^*−/−*^ MEFs to below wild-type levels (Fig. 5a). MEFs lacking a functional TSC have increased expression of a number of genes involved in metabolic pathways, including glycolysis, sterol, and lipid biogenesis, which can be reversed by treatment with rapamycin^61^. We find that MEFs lacking *Tsc1* have increased expression of *Pdk1*, *Pfkp*, *Mvk*, *Sc5d*, *Ascl3*, and *Scd1*, and that the expression of these genes can be repressed to normal levels by treatment with either 100 nM rapamycin or 100 nM DL001 (Fig. 5b–d). Importantly, rapamycin and DL001 are equally efficient at suppressing hyperactive mTORC1. Finally, as many of the symptoms of TSC1 such as seizures originate in the brain, we realized it was critically important to determine if DL001 can regulate mTORC1 signaling in the brain. We find that rapamycin and DL001 are both capable of efficiently inhibiting mTORC1 in the brain (Fig. 5e).

## Discussion

Here, we determined that DL001 is an effective mTORC1-specific rapalog both in cell culture and in vivo, and we demonstrate for the first time that the negative side effects of rapamycin and its analogs, including metabolic disruption and immunosuppression, may be avoided in whole or in part by a compound that specifically targets mTORC1. Importantly, as DL001 is an FKBP12-dependent rapalog, its effects on the rapamycin-resistant functions of mTORC1^62^ is minimal; work in genetic mouse models suggests that inhibition of the rapamycin-resistant functions of mTORC1 may be associated with side effects such as allergic asthma, hypoglycemia, seizures, and the ability to build muscle mass with exercise^63–66^. Importantly, DL001 efficiently represses hyperactive mTORC1 in cells lacking a functional TSC. As hyperactive mTORC1 drives much of the pathology of tuberous sclerosis and other mTORopathies^67^, DL001 and related molecules may be of clinical use in safely treating these disorders.

An important limitation of our study is that we have not conducted detailed pharmacokinetic studies to characterize the metabolism and elimination of DL001. While we have previously suggested that rapalogs that are eliminated more rapidly may have reduced (but still significant) side effects as a result of decreased mTORC2 inhibition^20^, the only parameter directly linked to the appearance of adverse events in clinical studies of rapalogs is the trough concentration^42,43^. We estimate that the half-life of DL001 is about 60% of that of rapamycin, with a near-trough concentration (*C*_min_) approximately 25% lower. Importantly, even this reduced near-trough blood level of DL001 is substantially higher than blood levels of rapamycin that we have previously demonstrated to be sufficient to inhibit mTORC2 in vivo^24,34,44^. The minor differences in blood levels of rapamycin and DL001 we have observed are therefore not adequate to explain the in vivo metabolic differences we have observed with respect to mTORC2-mediated side effects, particularly given the potent differences in mTORC2 specificity we observed between rapamycin and DL001 in cell culture. However, we note that the concentration of DL001 or its metabolism in different tissues may contribute to our observation that DL001 appears to more effectively suppress mTORC1 signaling in certain tissues than in others. As rapalogs are distributed extensively in peripheral tissues, including key metabolic tissues, conducting a full analysis of DL001 pharmacokinetics and metabolism in both blood and in specific tissues will be important to help advance our understanding of this molecule.

Important work remains to be completed prior to the clinical use of DL001 and related molecules, including pre-clinical studies in disease models, a direct assessment of the effects on immune system function, and a direct comparison of the side effects of DL001 or related molecules with rapalogs such as everolimus, which is FDA-approved to treat specific manifestations of TSC, in both male and female mice. While unanswered questions remain, including the impact of DL001 and related molecules on other rapamycin-associated side effects, the exact molecular mechanisms responsible for the reduced impact of DL001 on mTORC2 activity, and the ability of these molecules to delay age-related diseases and extend healthspan, our results demonstrate for the first time that highly selective pharmacological inhibition of mTORC1 can be achieved in vivo and that this avoids many of the side effects associated with long-term rapamycin treatment.

## Methods

### Materials

For western blotting, antibodies to phospho-AKT S473 (4060), AKT (4691), phospho-p70 S6 kinase (9234), p70 S6 kinase (2708), phospho-S6 ribosomal protein (2215 or 5364), S6 ribosomal protein (2217), p-4EBP1 S65 (9451), p-4EBP1 T37/S46 (2855), total 4EBP1 (9452), p-S757 ULK1 (14202), ULK1 (8054), mTOR (2792), RICTOR (2140), HSP90 (4877), β-tubulin (2128S), FKBP51 (12210S), and p-PKA substrate (9621) were from Cell Signaling Technology; β-actin (A2228) was from Sigma; ATGL (SC-365278) was from Santa Cruz; and FKBP12 (ab2918) was from Abcam. All antibodies for western blotting were used at a dilution of 1:1000, except that p-T389 S6K1 was used at a dilution of 1:500 for the experiments shown in Fig. 3e, f. For immunoprecipitation, antibody to RICTOR (A300-458A) was from Bethyl Laboratories and Protein G Agarose (PI20398) was from Fisher, with exact antibody amounts and concentrations as specified below. For flow cytometery, antibodies to CD4 (75-0041-U025, used at 0.2 µg µL^−1^) and CD8 (80-0081-U025, used at 0.5 µg µL^−1^) were from Tonbo; antibodies to CD25 (47-0251-80, used at 0.2 µg µL^−1^) and FOXP3 (25-5773-82, used at 0.2 µg µL^−1^) were from eBioscience, and antibodies to CD11a (553121, used at 0.2 µg µL^−1^), and CD3 (563204, used at 0.2 µg µL^−1^) were from BD. Protease and phosphatase inhibitor cocktail tablets were from Fisher. Other chemicals were purchased from Sigma unless noted. Glucose measurements were performed using a Bayer Contour blood glucose meter and test strips. Mouse Insulin ELISA kits were purchased from Crystal Chem. Rapamycin was purchased from LC Labs. 2.0 mL Tough Tubes with Caps (13119–500) and 1.4 mM ceramic beads (13113-325), were purchased from Mo-Bio Laboratories, Carlsbad, CA.

### DL001

The chemical purity and identity of DL001 was determined through Liquid chromatography–mass spectrometry, spectrographic analysis using ^1^H and ^13^C NMR, 2D Heteronuclear Multiple Bond Coherence (HMBC) and COSY Correlation NMR (Supplementary Fig. 7). DL001: ^1^H NMR (major trans rotamer, 600 MHz, DMSO-*d*_6_, 300 K) ppm 6.44 (d, *J* = 1.6 Hz, 1H), 6.39 (dd, *J* = 14.8, 11.0 Hz, 1H), 6.26–6.19 (m, 1H), 6.15–6.08 (m, 2H), 5.43 (dd, *J* = 14.8, 9.6 Hz, 1H), 5.11 (d, *J* = 4.3 Hz, 1H), 5.09 (br d, *J* = 10.1 Hz, 1H), 4.98 (ddd, *J* = 8.5, 4.2, 3.1 Hz, 1H), 4.95–4.92 (m, 1 H), 4.88 (d, *J* = 7.0 Hz, 1H), 4.42 (d, *J* = 4.4 Hz, 1H), 4.26 (dd, *J* = 7.0, 4.5 Hz, 1H), 4.04–3.97 (m, 1H), 3.95 (m, 1H), 3.71–3.59 (m, 1H), 3.46–3.40 (m, 1H), 3.34–3.11 (m, 3H), 3.05 (s, 3H), 2.77 (dd, *J* = 17.5, 2.7 Hz, 1H), 2.49–2.42 (m, 1H), 2.34 (dd, *J* = 17.7, 8.9 Hz, 1H), 2.28–2.16 (m, 2H), 2.13–2.08 (m, 1H), 2.07–1.99 (m, 1H), 1.89–1.75 (m, 4H), 1.74 (s, 3H), 1.69–0.80 (m, 33H), 0.79–0.70 (m, 5H); ^13^C NMR (*major trans rotamer* 150 MHz, DMSO-*d*_6_, 300K): ppm 213.5, 208.4, 199.3, 176.9, 169.7, 167.5, 139.9, 139.8, 138.3, 132.9, 130.9, 127.4, 124.8, 99.5, 82.7, 78.1, 76.5, 74.1, 69.7, 66.6, 55.9, 51.3, 45.7, 44.1, 40.6, 40.2, 39.9, 39.1, 35.8, 35.7, 35.5, 35.2, 33.8, 32.2, 30.8, 30.2, 26.9, 26.7, 25.0, 22.1, 21.0, 16.1, 15.2, 14.1, 13.8, 10.8; LC/MS (*m*/*z*): [M]^+^ calculated for C_49_H_75_NO_12_, 869.5 g mol^−1^; found 887.7 as [M + NH_4_]^+^ and 892.7 as [M + Na] ^+^.

### Ethical approval for animal research

All animal procedures were performed in complicance with institutional guidelines and all relevant ethical regulations for animal testing and research. Animal studies conducted at the Buck Institute for Research on Aging were approved by the Institutional Animal Care and Use Committee (IACUC) at the Buck Institute for Research on Aging, Novato, CA. Animal studies conducted at WSM VA Hospital were approved by the IACUC of the William S. Middleton Memorial Veterans Hospital, Madison, WI.

### Animals and treatments

All animal care and experimental procedures performed at the Buck Institute for Research on Aging were approved by the IACUC at the Buck Institute for Research on Aging. In these experiments, the results of which are described in Fig. 3a–d and Fig. S3, 12 10-week-old female C57BL/6J mice were given intraperitoneal injections of either 8 mg kg^−1^ rapamycin, 13 mg kg^−1^ DL001, or vehicle every other day for 3 weeks (4 mice/group). Following the last injection, mice were fasted overnight. Mice were injected with insulin (0.75 U kg^−1^) 15 min prior to dissection and tissues were immediately frozen in liquid nitrogen. Our analysis of mTORC1 and mTORC2 signaling in the tissues of vehicle and rapamycin-treated mice were previously reported^30^.

Animal studies conducted at WSM VA Hospital were approved by the IACUC of the William S. Middleton Memorial Veterans Hospital. In these experiments, the results of which are described in Figs. 3e, f and 4, and Figs. S4–S6, 45 C57BL/6J male mice were purchased from The Jackson Laboratory at 8 weeks of age and housed three per cage. All mice were fed LabDiet 5001, the standard facility chow. Following 1 week of acclimation to the facility, mice were weighed and the body composition of all mice was measured with an EchoMRI 3-in-1 system. Cages were sorted into three groups—vehicle, rapamycin, and nID390—such that the average weight was similar for all three groups. Rapamycin (LC Labs) and DL001 (Delos Pharmaceuticals) were dissolved in ethanol and diluted in filter-sterilized vehicle (5% Tween-80, 5% PEG-40, 0.9% NaCl) immediately prior to intraperitoneal injection. Vehicle, 8 mg kg^−1^ rapamycin or 12 mg kg^−1^ DL001 was administered every other day. Following the last injection, mice were fasted overnight for 16 h. Mice were injected with insulin (0.75 U kg^−1^) 15 min prior to dissection, and tissues were immediately frozen in liquid nitrogen.

### Immunoblotting and immunoprecipitation

Frozen tissues collected at the Buck Institute were homogenized using the Omni TH homogenizer (Omni International) on ice in RIPA buffer (300 mM NaCl, 1.0% NP-40, 0.5% sodium deoxycholate, 0.1% sodium dodecyl sulfate (SDS), 50 mM Tris (pH 8.0), Protease inhibitor cocktail (Roche), phosphatase inhibitor 2, 3 (Sigma)), and then centrifuged at 17,000 *g* for 15 min at 4 °C. The supernatants were collected and protein concentration was determined using the DC protein assay (Biorad). Equal amounts of protein were resolved by SDS polyacrylamide gel electrophoresis (SDS-PAGE) and transferred to nitrocellulose membrane using the Invitrogen Nu-Page system (Carlsbad, CA). Western blot analysis was then performed.

Frozen tissues collected at UW-Madison, as well as cells and tissues, were lysed in cold RIPA buffer supplemented with phosphatase inhibitor and protease inhibitor cocktail tablets (PI88669; Fisher Scientific). Tissues for western blotting other than brain were lysed in RIPA buffer using a FastPrep 24 (M.P. Biomedicals) with bead-beating tubes and ceramic beads (Mo-Bio Laboratories), and then centrifuged^28^. Protein concentration was determined by Bradford (Pierce Biotechnology). Twenty micrograms protein was separated by SDS-PAGE on 8%, 10%, or 16% resolving gels (Thermo Fisher Scientific, Waltham, MA). Proteins were then transferred to PVDF membrane (Millipore). Immunoprecipitation of RICTOR was performed using flash-frozen liver lysed in cold CHAPS buffer^31,68^. Following protein lysis and determination of protein concentration, 1 mg of protein lysate in 500 µL (2 µg µL^−1^) was precleared with 20 µL of protein G agarose beads, and then incubated with 2 µL of RICTOR antibody (1:250) overnight. Following antibody incubation, 25 µL of protein G agarose beads were added and incubated at 4 °C for 3 h. Beads were then washed to removed unbound protein, and boiled in 2× loading buffer prior to SDS page analysis. Imaging was performed using a GE ImageQuant LAS 4000 imaging station. Brain tissue was analyzed by Aeonian Pharmaceuticals using a similar protocol and imaging was performed using a Biorad ChemiDoc MP imager.

Raw western blot images are provided in the Source Data file. Quantification of all blots was performed by densitometry using NIH ImageJ software. Approximate molecular weights, based on the expected molecular weight and verified using molecular weight standards run on each gel, are indicated to the right of each immunoblot.

### AlphaLISA assay

PC3 cells were maintained in F12K media (ATCC/GIBCO, Cat# ATCC 30-2004) supplemented with additional 10% fetal bovine serum (FBS) (Gemini, cat# 100–106), 1% Penicillin/Streptomycin (Life Technologies, cat # 15140–122), and 2 mM l-glutamine (Life Technologies, cat# 25030) and cultured at 37 °C under an atmosphere of 95% air and 5% CO_2_. For AlphaLISA experiments, cells were seeded in 96-well plates for 24 h and treated at various concentrations of rapamycin or DL001 (from approx. 8 fM to 10 μM) for 24 h using two methods: for examination of mTORC1 signaling, the assay was performed in the continuous presence of serum; for examination of mTORC2 signaling, cells were (1) treated with the compounds for 8 h in growth media containing 10% serum; (2) the media was then replaced by serum-free media containing the respective drug concentration and incubated for an additional 16 h; and (3) immediately prior to cell lysis, cells were treated with 12% serum for 15 min Cells were harvested by lysis in the buffer supplied with the AlphaLISA kit. mTORC1 inhibition was then determined using the AlphaLISA SureFire kit for Phospho-S6 Ribosomal Protein (Ser240/244) (TGRS6P2S500; Perkin Elmer). mTORC2 inhibition was determined by the AlphaLISA SureFire kit for AKT 1/2/3 (S473) (TGRA4S500; Perkin Elmer). Cells from plates were lysed using 50 μL of the lysis buffer and incubated for 10 min at room temperature while shaking. Four microliters of cell lysates were then analyzed for 2 h at room temperature according to the manufacturer’s protocol. The donor mix was then added and the resulting solution was incubated for 2 h at room temperature. AlphaLISA signal was read on a Fusion-Alpha FP HT (Perkin Elmer). Percent inhibition was calculated by comparison to the highest inhibition value obtained in the response–concentration curve. IC_50_s were calculated using Prism software. All IC_50_ experiments were conducted in triplicates with rapamycin and vehicle controls.

### Cell culture and shRNA knockdown

PC3 (CRL-1435), AML12 (CRL-2254), and HepG2 (HB-8065) cells were purchased from the American Type Culture Collection (ATCC). PC3 cells transduced with lentivirus expressing shRNA against *FKBP12* or *FKBP51* or a nonspecific control were thawed from Kennedy laboratory stocks^30^ and reselected in puromycin prior to experimentation. PC3 and HepG2 cells were cultured in DMEM (Fisher Scientific, MT10013CV) containing 10% FBS (Sigma, 12303C) and 1% penicillin–streptomycin (Fisher Scientific, MT30002CI). AML12 cells were cultured in DMEM/F-12 media (Fisher Scientific, MT10090CV) with 10% FBS and 1% penicillin–streptomycin.

### Mouse embryonic fibroblast cell culture and qPCR

Immortalized mouse embryonic fibroblasts lacking *Tsc1*, and paired *Tsc1*^*+/+*^ cells, were the kind gift of Dr. David Kwiatkowski, and were grown in DMEM containing 10% FBS. For signaling and gene expression experiments, cells were cultured for 24 h in serum-free DMEM containing the specified concentration of rapamycin or DL001, or an equal volume of DMSO (vehicle). For gene expression, cells were harvested and RNA was extracted, cDNA was synthesized, and qPCR was performed^69^. Mouse qPCR primer sequences for the genes examined have been utilized previously^61^ and are listed here: Pdk1: F: ggcggctttgtgatttgtat, R: acctgaatcgggggataaac; Pfkp: F: aggagggcaaaggagtgttt, R: ttggcagaaatcttggttcc; Mvk: F: gggacgatgtcttccttgaa, R: gaacttggtcagcctgcttc; Sc5d: F: ccaaatggctggattcatct, R: gtccacagggtgaaaagcat; Ascl3: F: ggggctggaacaattacaga, R: atagccaccttcctcccagt; Scd1: F: ctgacctgaaagccgagaag, R: gcgttgagcaccagagtgta.

### Tolerance tests and blood collection

Mice were fasted overnight for 16 h and then injected with either 1 g kg^−1^ glucose or 2 g kg^−1^ pyruvate. For glucose and pyruvate tolerance tests, small blood samples were taken from a tail vein nick at time intervals and read using a Bayer Contour blood glucose meter and test strips. For determination of fasting glucose and insulin, blood glucose levels were read using a glucometer and then 50 μL of blood was collected into a EDTA tube immediately prior to and 15 min following glucose administration. Insulin levels were determined using a Mouse Insulin ELISA kit (Crystal Chem).

### Splenocyte preparation and flow cytometry

Splenocytes were prepared following a procedure from Life Technologies. Briefly, spleens were collected in a buffer containing PBS, 0.1% bovine serum albumin (BSA), and 0.6% Na-citrate, and then macerated through a 70 μM filter using a syringe plunger. Following centrifugation at 300*g* for 10 min, cells were resuspended in the same buffer, and recentrifuged. The splenocytes were suspended in PBS and 0.1% BSA with Ca^2+^ and Mg^2+^, and incubated with approximately 100 units of DNAase (Sigma). Splenocytes were then filtered through a 40 μM filter and red blood cells were lysed. Splenocytes were then centrifuged, suspended in PBS with 0.1% BSA, and brought to the UWCCC Flow Cytometry Lab for immunostaining and flow cytometry on a BD LSRII (San Jose, California). Data were collected using BD FACSDiva, Version 8.0 and analyzed with FlowJo X, Version 10.0.7r2 (FlowJo, LLC, Ashland, OR). An example of the gating strategy used to analyze the flow cytometry data is shown in Supplementary Fig. 8.

### Proteomics

PC3 cells were grown in 10% DMEM with FBS and treated with 100 nM rapamycin or 100 nM DL001 for 24 h. Cells were washed with cold PBS and harvested in 8 M Urea buffer (8 M Urea, 40 mM NaCl, 50 mM tris, 2 mM MgCl_2_, 50 mM NaF, 50 mM b-glyceradelhyde phosphate, 1 mM sodium orthovanadate, 10 mM sodium pyrophosphate) with addition of protease and phosphatase inhibitor (Thermo Fisher Scientific). Cells were then sonicated and supernatant was collected following centrifugation. Samples were then prepared for quantitative proteomic analysis by the Proteomics Facility of the University of Wisconsin Biotechnology Center. Briefly, peptides from each of the 4 Vehicle, 3 Rapamycin-, and 3 DL001-treated samples were digested with trypsin, the peptides were derivatized separately with isobaric mass tags (TMT-10plex; ThermoFisher Scientific), and the samples were pooled. Following offline fractionation, four high-pH fractions were analyzed using shallow, 4-h gradients by nanoflow HPLC on an Orbitrap Elite (ThermoFisher Scientific). Database searching and quantitation was performed using Proteome Discoverer 2.2 (ThermoFisher Scientific). Peptides with significant differences between treatment groups were identified using the limma R package^70^ and the resultant *p* values were then FDR-adjusted using *q* values. Differentially affected KEGG and Reactome categories were identified by analyzing significantly altered proteins (*q* < 0.05) using gProfiler .

### Statistics

Statistical analysis was conducted using Prism 7 (GraphPad Software). Significance was tested by a two-sided Student's *t*-test for two-group comparisons or ANOVA followed by a two-sided Dunnett’s, Sidak, or Tukey post hoc test as specified in the figure legends for comparisons of three or more groups. Statistical analysis of proteomics data was conducted in R (version 3.5.0).

### Reporting Summary

Further information on research design is available in the Nature Research Reporting Summary linked to this article.

## Supplementary information

Supplementary Information

Reporting Summary

Source Data

## Data Availability

The source data underlying all figures are provided as a Source Data file. Proteomics data has been deposited in MassIVE with accession code MSV000083602 (doi:10.25345/C5TW5T). DL001 will be available at cost and subject to an MTA by Aeonian Pharmaceuticals or contracted non-commercial third party providers.
