# Supplementary material for: A novel rapamycin analog is highly selective for mTORC1 in vivo
Source: Nat Commun. 2019 Jul 19;10:3194. doi: 10.1038/s41467-019-11174-0 (PMC6642166; doi:10.1038/s41467-019-11174-0)
Supplement: Supplementary file 1 — Supplementary Information [file 41467_2019_11174_MOESM1_ESM.pdf]

A novel rapamycin analog is highly selective for mTORC1 *in vivo*

Schreiber KH, Arriola Apelo SI, *et al*.

Supplementary Figures

Supplementary Figures

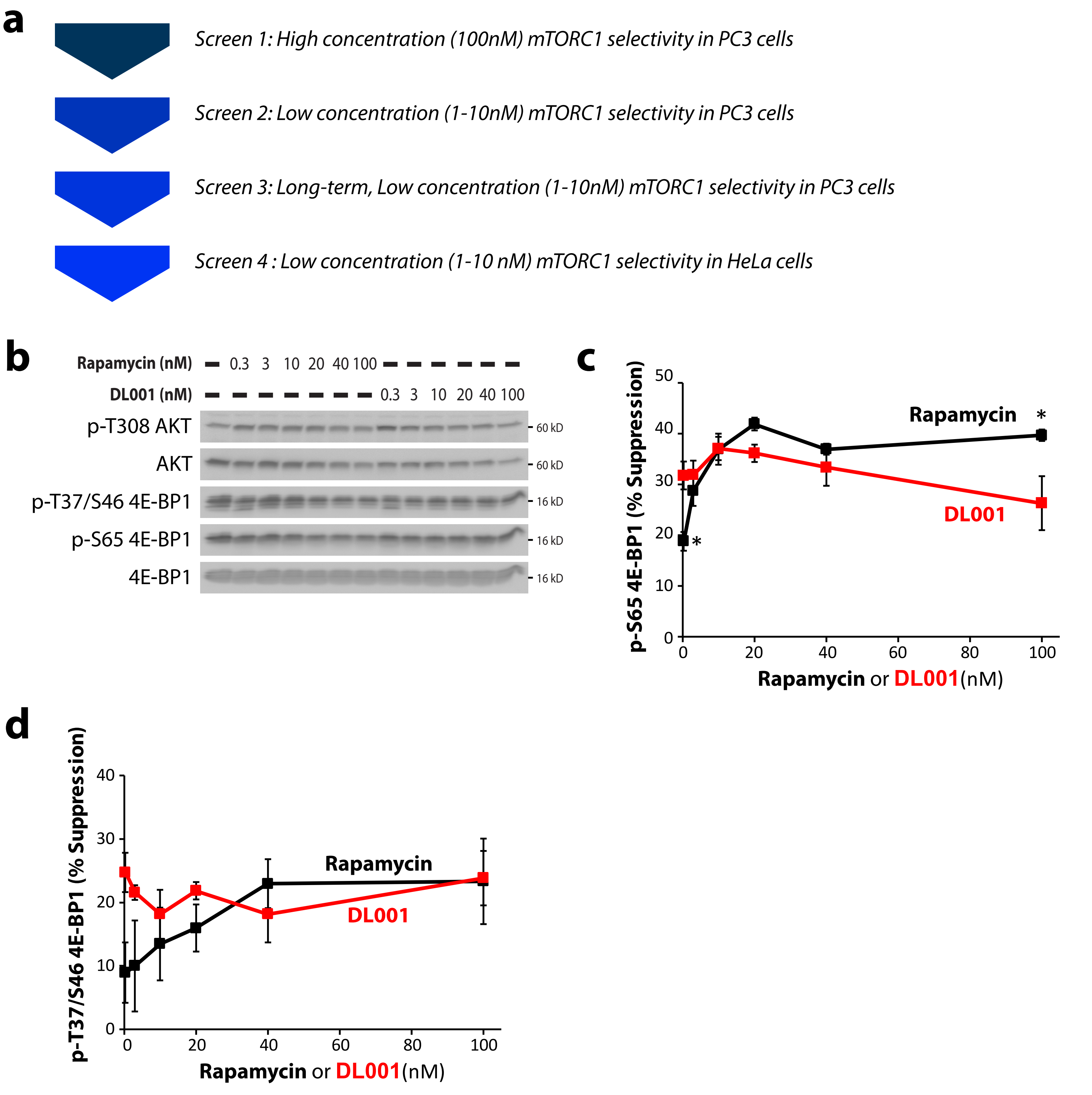

Supplementary Figure 1. Identification and characterization of DL001. (**a**) Approximately 90 rapamycin analogs were screened using a series of *in vitro* assays to assess the potency of mTORC1 and mTORC2 inhibition. (**b**) PC3 cells were treated with rapamycin or DL001 at 0.3-100 nM for 48 hours and the phosphorylation of 4E-BP1 at T37/S46 and S65, and the phosphorylation of AKT T308 was determined by Western blotting. (**c-d**) The inhibition of (**c**) 4E-BP1 S65 phosphorylation and (**d**) 4E-BP1 T37/S46 phosphorylation was plotted (n=3 biologically independent experiments; * = $p < 0.05$, Sidak's test following two-way ANOVA). Error bars represent standard error. Source data are provided as a Source Data file.

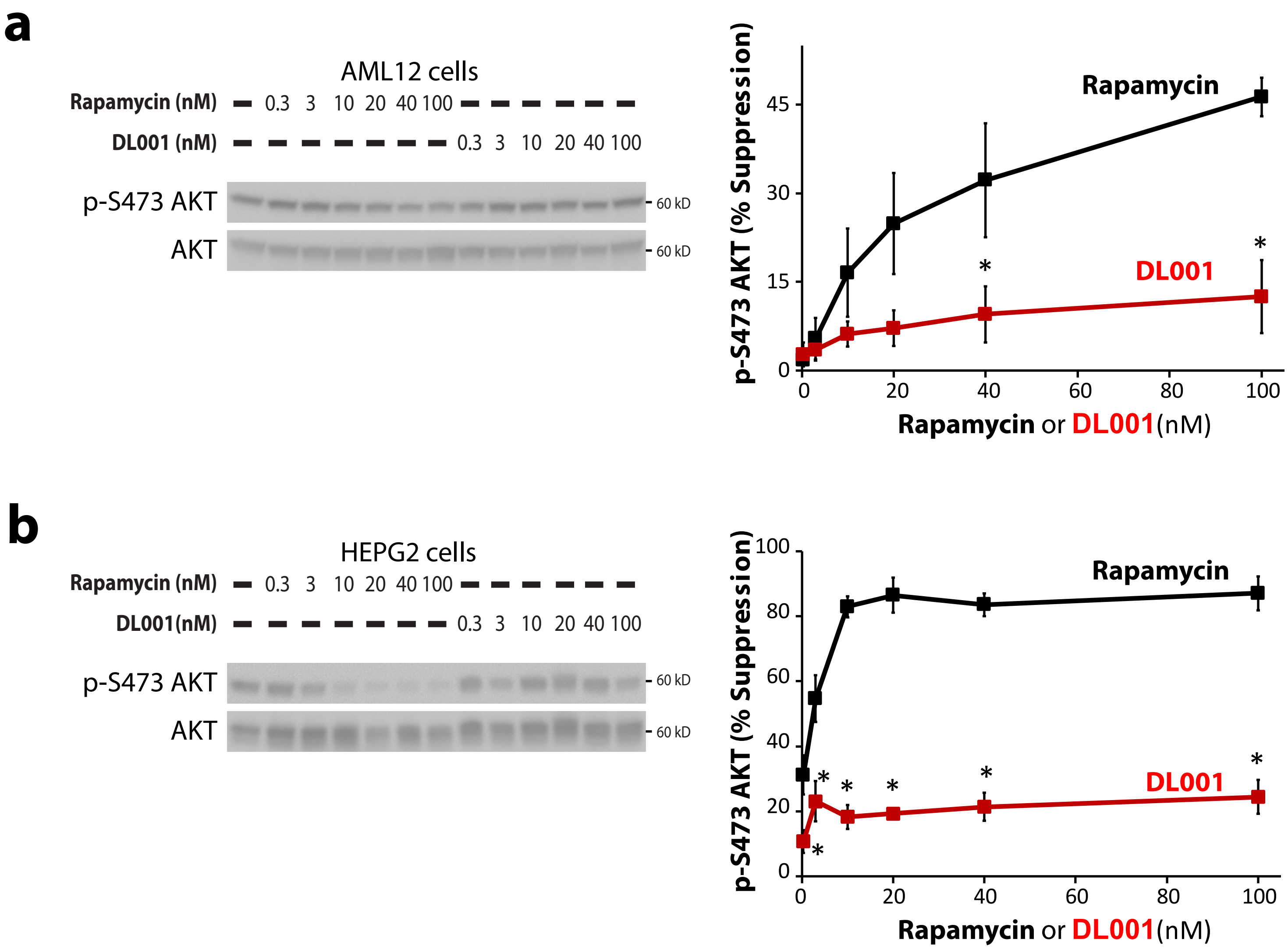

Supplementary Figure 2. DL001 has reduced activity on mTORC2 vs. rapamycin in multiple cell types. (**a-b**) (**a**) AML12 and (**b**) HepG2 cells were treated with 0.3 to 100nM rapamycin or DL001 for 48 hours and the phosphorylation of AKT S473 (a mTORC2 substrate) was determined by Western blotting; the inhibition of AKT S473 phosphorylation was plotted (n=3 biologically independent experiments; * = $p < 0.05$, Sidak's test following two-way ANOVA). Error bars represent standard error. Source data are provided as a Source Data file.

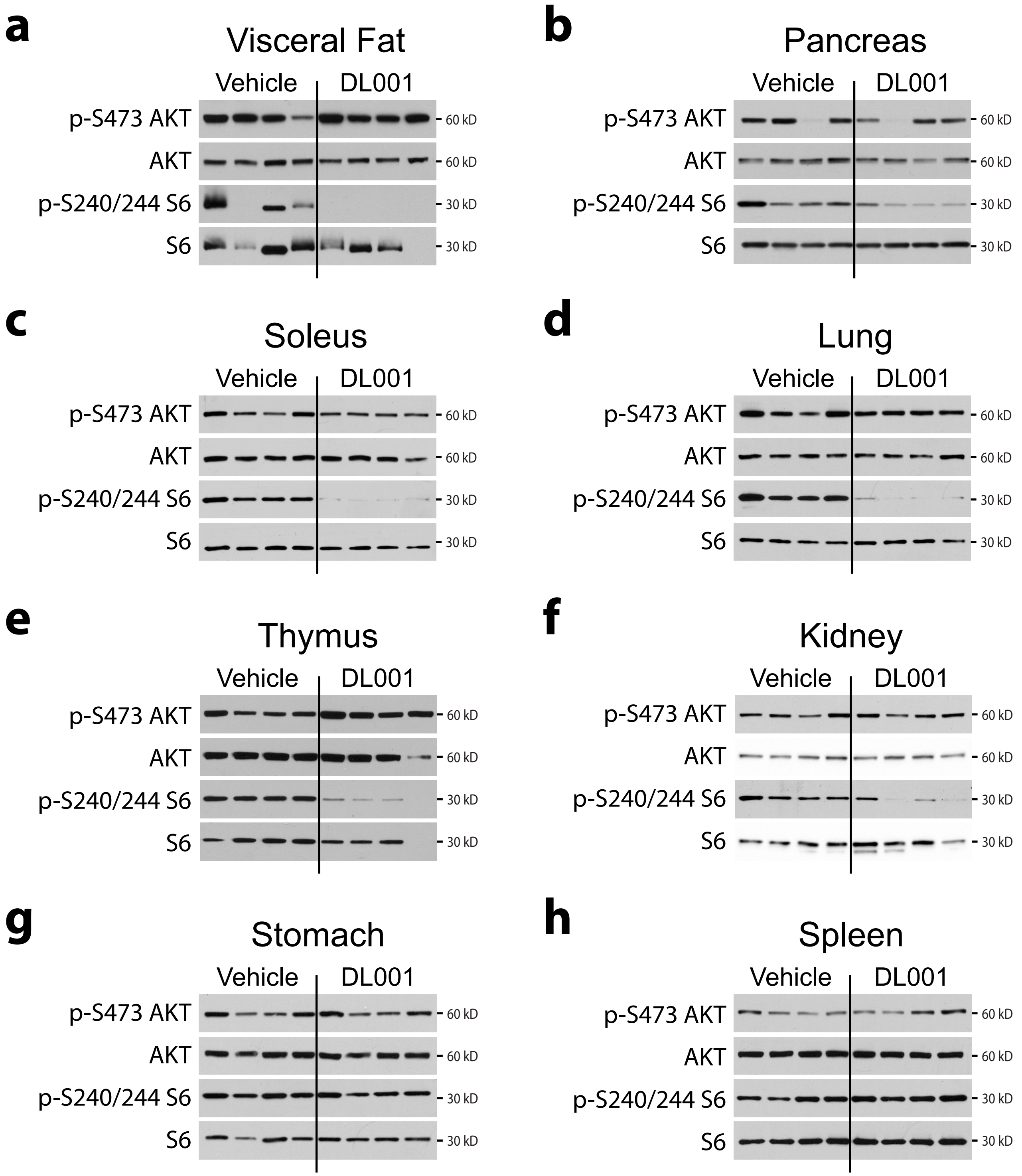

Supplementary Figure 3. DL001 specifically inhibits mTORC1 in most tissues. (**a-h**) C57BL/6J female mice treated every other day with 13 mg kg^{-1} DL001 were sacrificed and protein lysates were prepared from (**a**) visceral fat, (**b**) pancreas, (**c**) soleus, (**d**) lung, (**e**) thymus, (**f**) kidney, (**g**) stomach, and (**h**) spleen. Western blotting was then performed S240/S244 S6 (a readout for mTORC1 activity) and AKT S473 (a mTORC2 substrate). Source data are provided as a Source Data file.

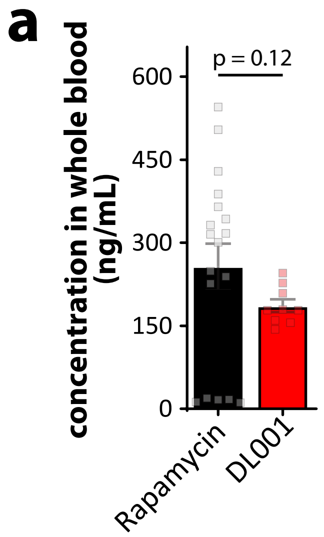

Supplementary Figure 4. Blood levels of rapamycin and DL001. (**a**) blood levels of Rapamycin (dosed at 8 mg kg^{-1}) and DL001 (dosed at 12 mg kg^{-1}) were determined approximately 16 hours after the final administration of the compounds (n = 9 biologically independent animals per group, * = $p < 0.05$, t-test). Error bars represent standard error. Source data are provided as a Source Data file.

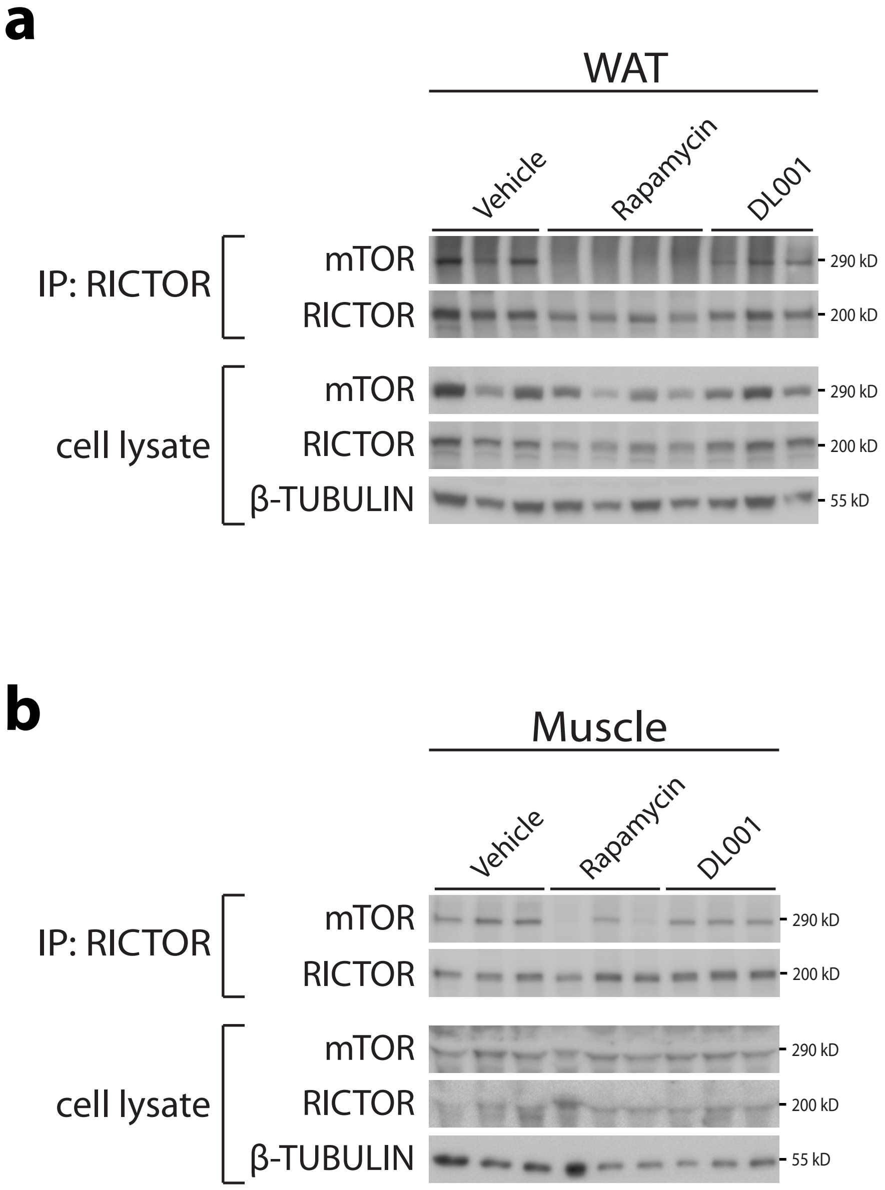

Supplementary Figure 5. Disruption of mTORC2 *in vivo* by rapamycin, but not by DL001. (**a**, **b**) Effect of 8 mg kg^{-1} rapamycin or 12 mg kg^{-1} DL001 administered every other day on the integrity of mTORC2 . RICTOR was immunoprecipitated from (**a**) white adipose tissue and (**b**) skeletal muscle, followed by immunoblotting for mTOR and RICTOR; immunoblots of cell lysate were performed as a control. Source data are provided as a Source Data file.

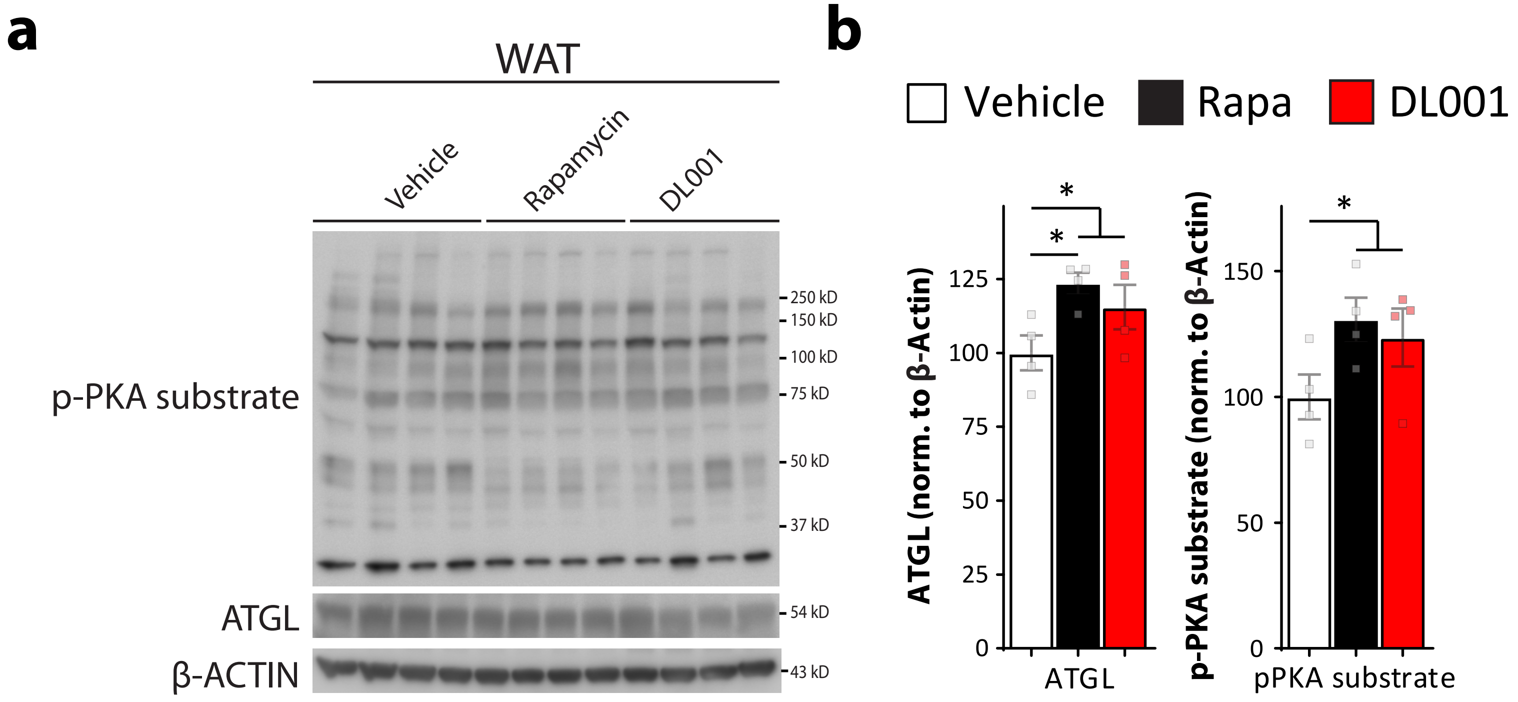

Supplementary Figure 6. Inhibition of mTORC1 increases lipolysis in white adipose tissue (a) White adipose tissue from C57BL/6J mice treated every other day with either vehicle, 8 mg kg^{-1} rapamycin, or 12 mg kg^{-1} DL001 was immunoblotted using an antibody recognizing the PKA substrate motif, ATGL, and β-actin. (**b**) Quantification of ATGL expression and phosphorylated PKA motifs (n = 4 biologically independent animals per group; for comparison of three groups, * = $p < 0.05$, Holm-Sidak's test following ANOVA; for comparison of two groups, * = $p < 0.05$, t-test). Error bars represent standard error. Source data are provided as a Source Data file.

Supplementary Figure 7

A.

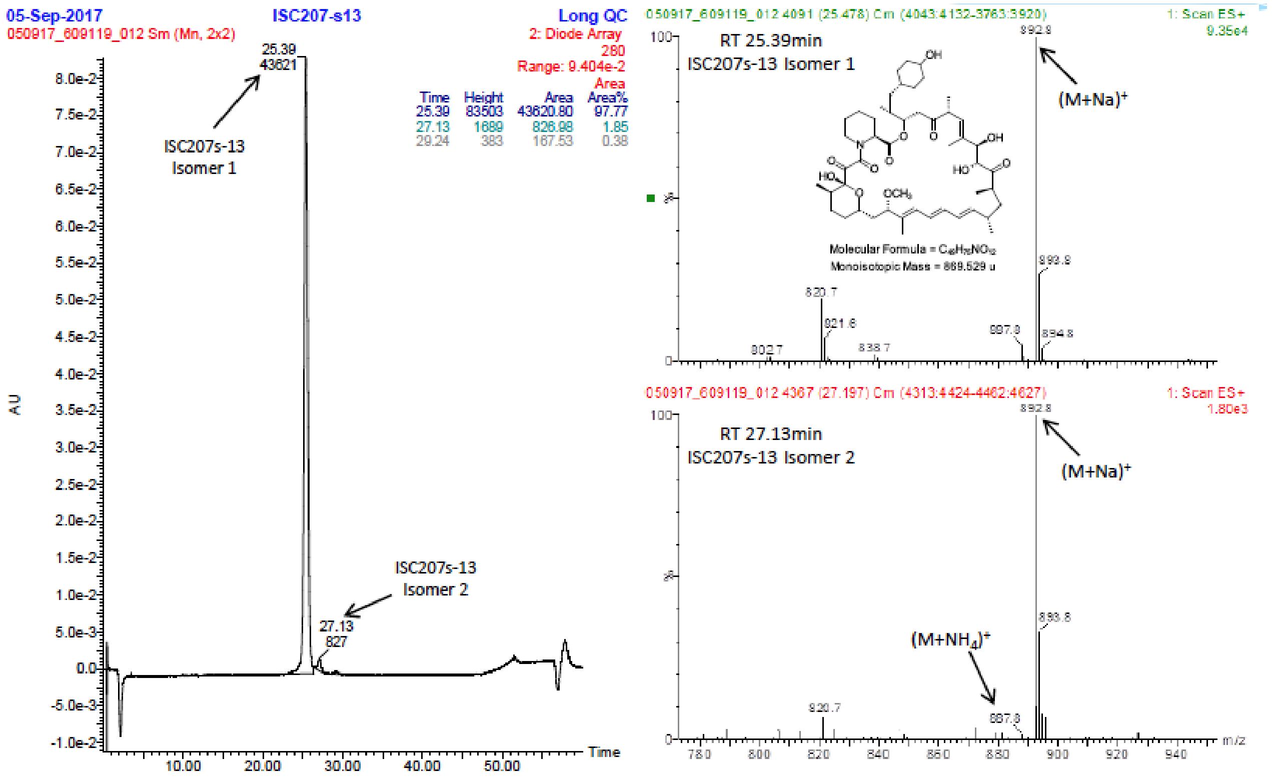

Identity

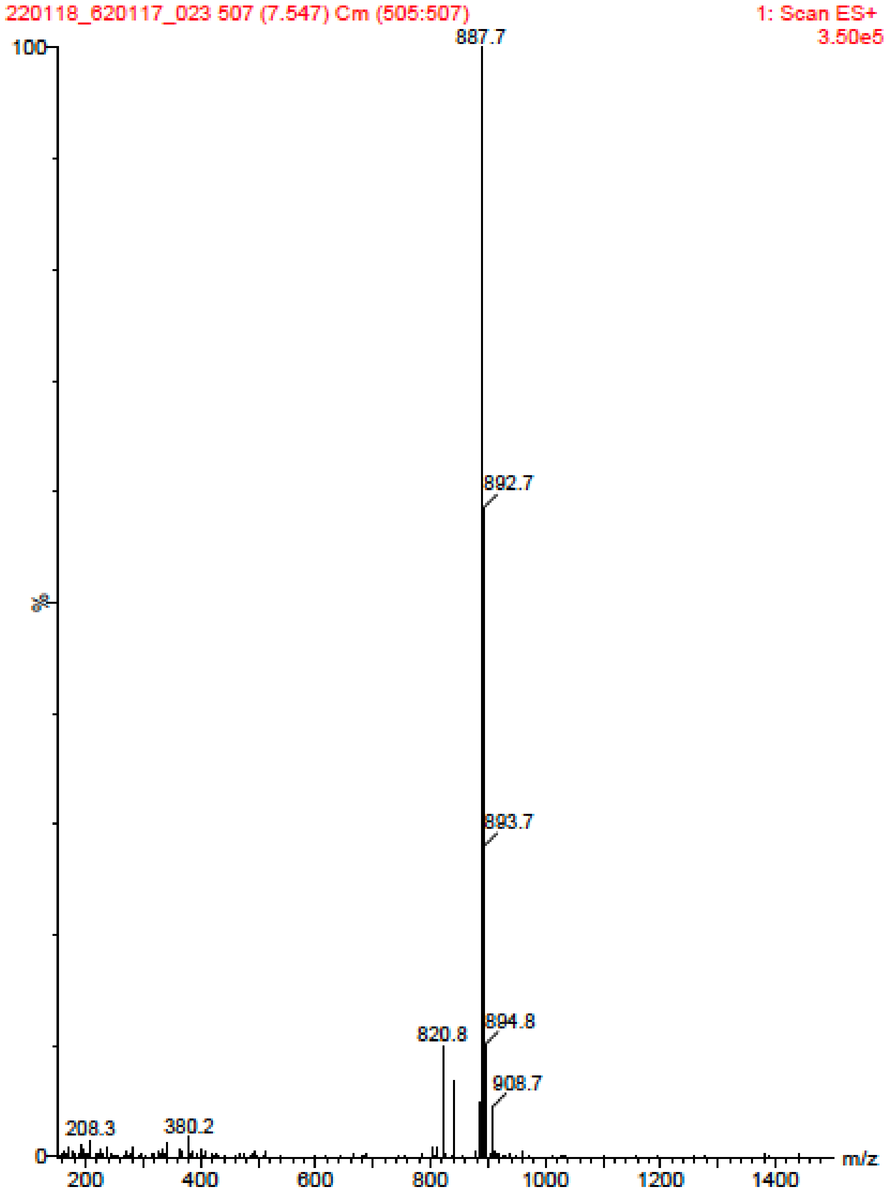

Molecular Formula	$C_{49}H_{75}NO_{12}$
Exact Mass (calculated)	869.5 g.mol^{-1}

m/z observed and species	887.7 as $[M+NH_4]^+$ and 892.7 as $[M+Na]^+$
Molecular Weight (of salt if applicable)	870.1 g.mol^{-1}

B.

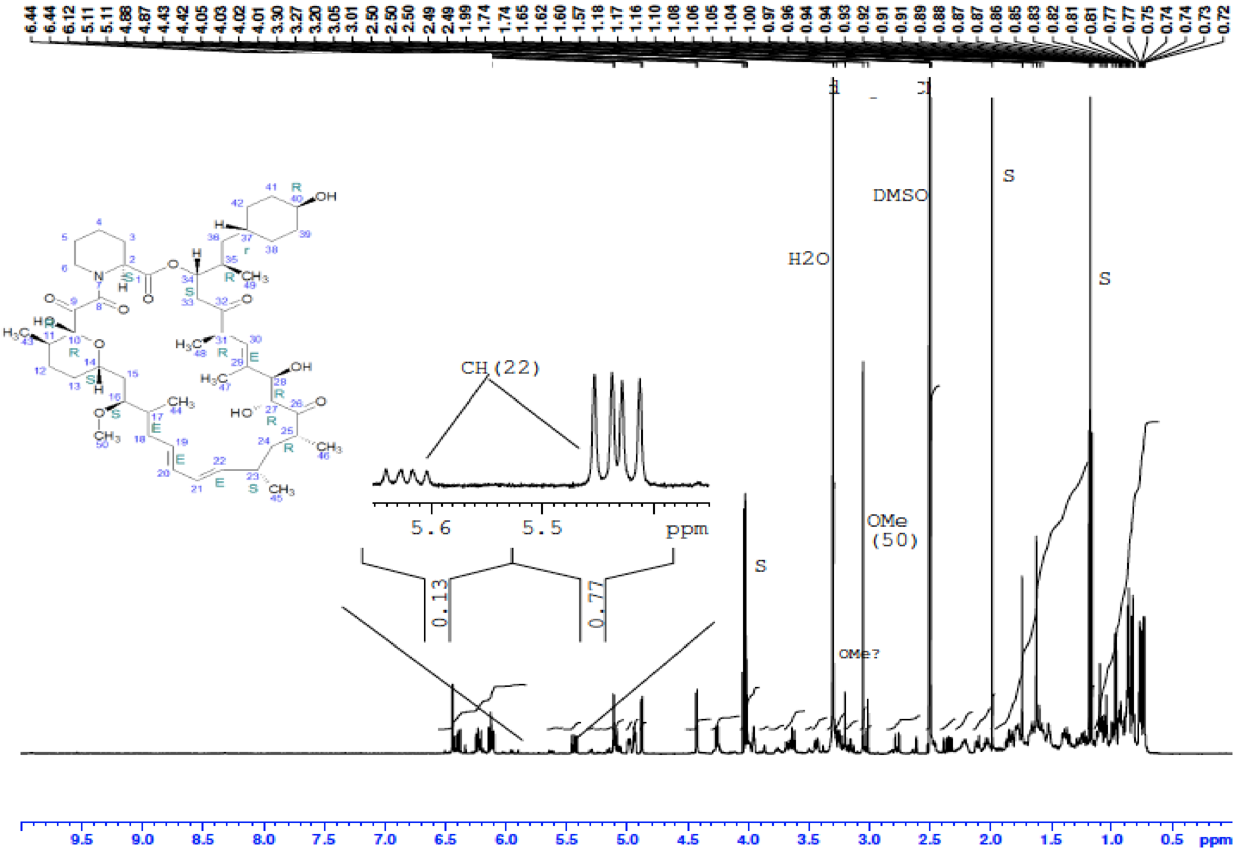

^{1}H-NMR Structural assignments

Chemical structure: C49H75N012

The compound exists as two rotamers at a Trans/Cis ratio of 85/15

^{1}H NMR (*major trans rotamer*, 600 MHz, DMSO-d_6, 300K): ppm 6.44 (d, *J*=1.6 Hz, 1 H), 6.39 (dd, *J*=14.8, 11.0 Hz, 1 H), 6.26 - 6.19 (m, 1 H), 6.15 - 6.08 (m, 2 H), 5.43 (dd, *J*=14.8, 9.6 Hz, 1 H), 5.11 (d, *J*=4.3 Hz, 1 H), 5.09 (br d, *J*=10.1 Hz, 1 H), 4.98 (ddd, *J*=8.5, 4.2, 3.1 Hz, 1 H), 4.95 - 4.92 (m, 1 H), 4.88 (d, *J*=7.0 Hz, 1 H), 4.42 (d, *J*=4.4 Hz, 1 H), 4.26 (dd, *J*=7.0, 4.5 Hz, 1 H), 4.04 - 3.97 (m, 1 H), 3.95 (m, 1 H), 3.71 - 3.59 (m, 1 H), 3.46 - 3.40 (m, 1 H), 3.34 - 3.11 (m, 3 H), 3.05 (s, 3 H), 2.77 (dd, *J*=17.5, 2.7 Hz, 1 H), 2.49 - 2.42 (m, 1 H), 2.34 (dd, *J*=17.7, 8.9 Hz, 1 H), 2.28 - 2.16 (m, 2 H), 2.13 - 2.08 (m, 1 H), 2.07 - 1.99 (m, 1 H), 1.89 - 1.75 (m, 4 H), 1.74 (s, 3 H), 1.69 - 0.80 (m, 33 H), 0.79 - 0.70 (m, 5 H)

C.

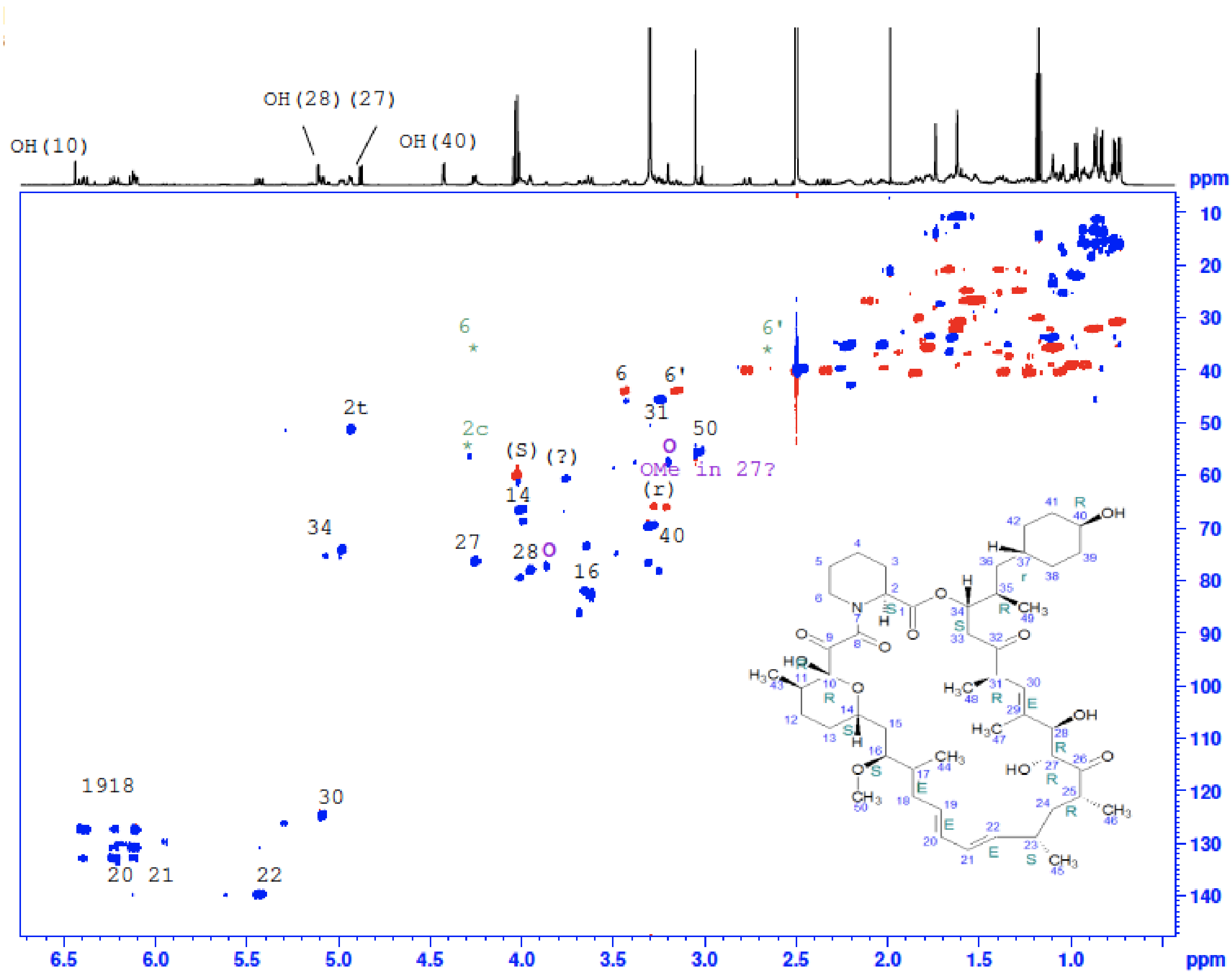

Structural assignments

Chemical structure: C49H75N012

^{13}C NMR (*major trans rotamer* 150 MHz, DMSO-d_6, 300K): ppm 213.5, 208.4, 199.3, 176.9, 169.7, 167.5, 139.9, 139.8, 138.3, 132.9, 130.9, 127.4, 124.8, 99.5, 82.7, 78.1, 76.5, 74.1, 69.7, 66.6, 55.9, 51.3, 45.7, 44.1, 40.6, 40.2, 39.9, 39.1, 35.8, 35.7, 35.5, 35.2, 33.8, 32.2, 30.8, 30.2, 26.9, 26.7, 25.0, 22.1, 21.0, 16.1, 15.2, 14.1, 13.8, 10.8

D.

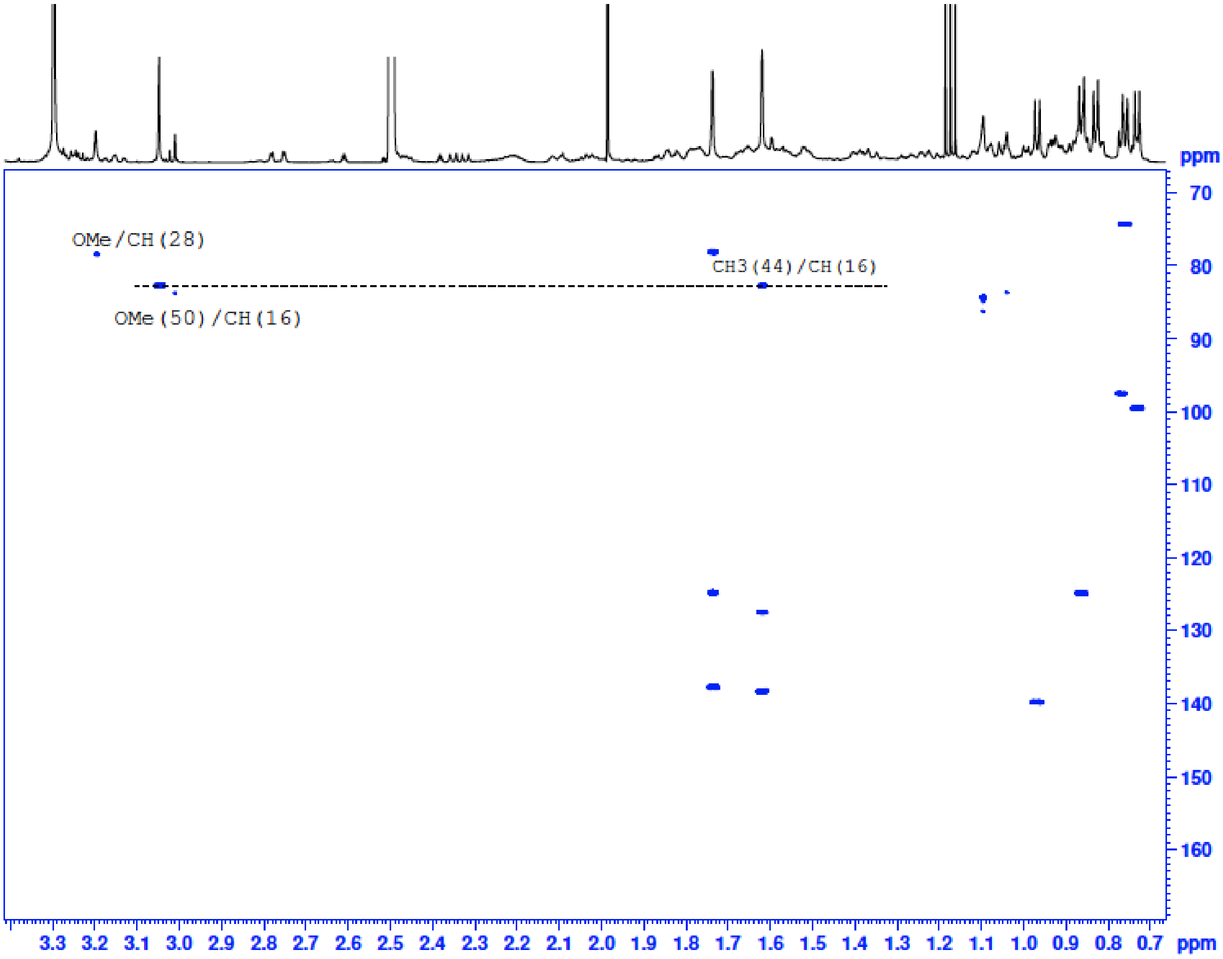

OMe/CH(28)
CH3(44)/CH(16)
OMe(50)/CH(16)
ppm
70
80
90
100
110
120
130
140
150
160
3.3 3.2 3.1 3.0 2.9 2.8 2.7 2.6 2.5 2.4 2.3 2.2 2.1 2.0 1.9 1.8 1.7 1.6 1.5 1.4 1.3 1.2 1.1 1.0 0.9 0.8 0.7 ppm

E.

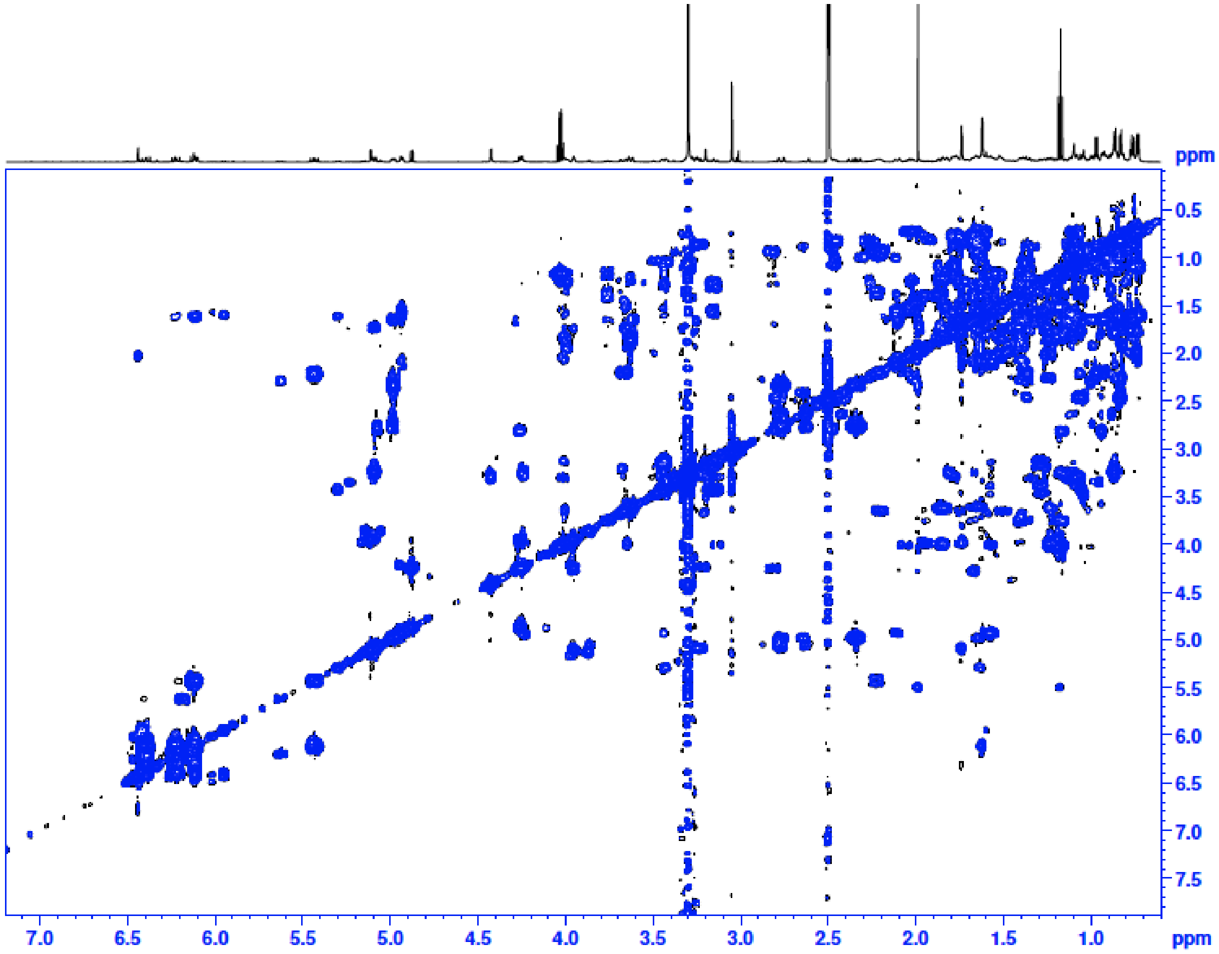

Supplementary Figure 7. Characterization of DL001. (**a**) LC/MS was used to determine the purity and identify of DL001. (**b-e**) The structure of DL001 was determined by (**b**) ^{1}H-NMR, (**c**) 2D Heteronuclear Single Quantum Coherence (HSQC) NMR, (**d**) 2D Heteronuclear Multiple Bond Correlation (HMBC) NMR, and (**e**) COSY Correlation NMR.

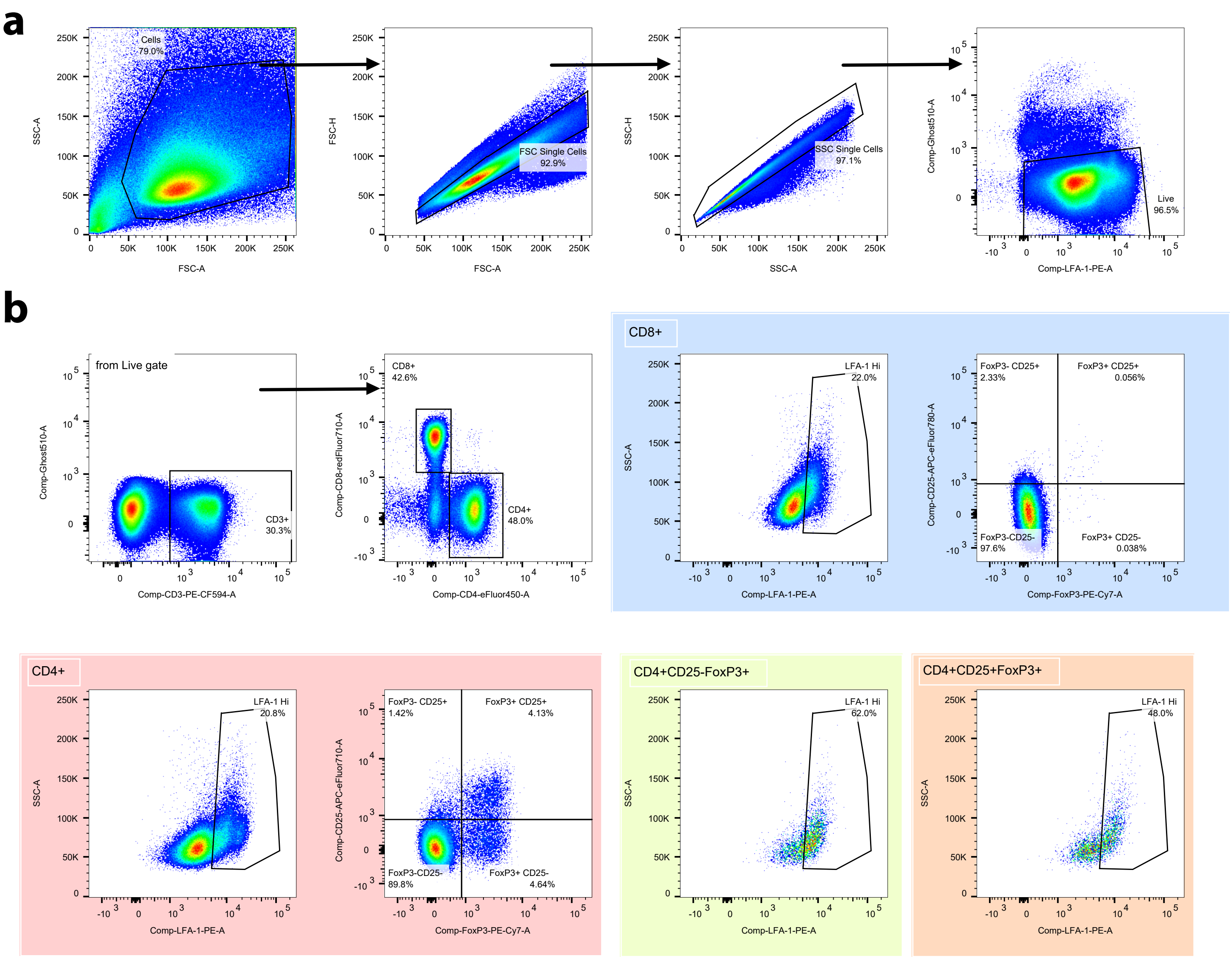

Supplementary Figure 8. Example of the staining and gating strategy used to analyze mouse splenocytes. Mouse splenocytes were stained with the complete panel. Fluorescence minus one (FMO) controls were used to define gates for CD25, FoxP3 and LFA-1. (**a**) Initial gating was done on SSC-A and FSC-A to identify main cell population followed by gating on FSC-H and FSC-A and then SSC-H and SSC-A to identify single cells. Dead cells were excluded by an amine reactive dye, Ghost510-A. (**b**) $CD3^+$ Lymphocytes were gated using Ghost510-A and CD3-PE-CF594-. Subsequent gating discriminated $CD4^+$ T cells and $CD8^+$ T Cells. Within $CD8^+$ T cells, all cells were LFA-1^+ using the FMO control to gate, so LFA-1 “Hi” cells were gated. CD25 and FoxP3 gates were set using FMO controls. Within $CD4^+$ T cells, all cells were LFA-1^+ using the FMO control to gate, so LFA-1 “Hi” cells were gated. CD25 and FoxP3 gates were set using FMO controls. LFA-1 “Hi” cells were gated within the $CD4^+CD25^+FoxP3^-$ gate and the $CD4^+CD25^+FoxP3^+$ gate.
