# Supplementary material for: A novel rapamycin analog is highly selective for mTORC1 in vivo
Source: Nat Commun. 2019 Jul 19;10:3194. doi: 10.1038/s41467-019-11174-0 (PMC6642166; doi:10.1038/s41467-019-11174-0)
Supplement: Supplementary file 2 — Reporting Summary [file 41467_2019_11174_MOESM2_ESM.pdf]

nature research

Corresponding author(s): Lamming DW

Last updated by author(s): May 26, 2019

Reporting Summary

Nature Research wishes to improve the reproducibility of the work that we publish. This form provides structure for consistency and transparency in reporting. For further information on Nature Research policies, see Authors & Referees and the Editorial Policy Checklist.

Statistics

For all statistical analyses, confirm that the following items are present in the figure legend, table legend, main text, or Methods section.

n/a	Confirmed	
☐	☒	The exact sample size (*n*) for each experimental group/condition, given as a discrete number and unit of measurement
☐	☒	A statement on whether measurements were taken from distinct samples or whether the same sample was measured repeatedly
☐	☒	The statistical test(s) used AND whether they are one- or two-sided<br>*Only common tests should be described solely by name; describe more complex techniques in the Methods section.*
☒	☐	A description of all covariates tested
☐	☒	A description of any assumptions or corrections, such as tests of normality and adjustment for multiple comparisons
☒	☐	A full description of the statistical parameters including central tendency (e.g. means) or other basic estimates (e.g. regression coefficient) AND variation (e.g. standard deviation) or associated estimates of uncertainty (e.g. confidence intervals)
☒	☐	For null hypothesis testing, the test statistic (e.g. *F*, *t*, *r*) with confidence intervals, effect sizes, degrees of freedom and *P* value noted<br>*Give P values as exact values whenever suitable.*
☒	☐	For Bayesian analysis, information on the choice of priors and Markov chain Monte Carlo settings
☒	☐	For hierarchical and complex designs, identification of the appropriate level for tests and full reporting of outcomes
☒	☐	Estimates of effect sizes (e.g. Cohen's *d*, Pearson's *r*), indicating how they were calculated

Our web collection on statistics for biologists contains articles on many of the points above.

Software and code

Policy information about availability of computer code

Data collection	No software was used
Data analysis	Statistical analysis was conducted using Prism 7/8 (GraphPad Software), except for analysis of proteomics data which was conducted in R (version 3.5.0).

For manuscripts utilizing custom algorithms or software that are central to the research but not yet described in published literature, software must be made available to editors/reviewers. We strongly encourage code deposition in a community repository (e.g. GitHub). See the Nature Research guidelines for submitting code & software for further information.

Data

Policy information about availability of data

All manuscripts must include a data availability statement. This statement should provide the following information, where applicable:

- Accession codes, unique identifiers, or web links for publicly available datasets
- A list of figures that have associated raw data
- A description of any restrictions on data availability

The source data underlying all figures are provided as a Source Data file. Proteomics data has been deposited in MassIVE with accession code MSV000083602 (doi:10.25345/C5TW5T). DL001 will be available at cost and subject to an MTA by Aeonian Pharmaceuticals or contracted non-commercial third party providers.

Field-specific reporting

Please select the one below that is the best fit for your research. If you are not sure, read the appropriate sections before making your selection.

☒ Life sciences ☐ Behavioural & social sciences ☐ Ecological, evolutionary & environmental sciences

For a reference copy of the document with all sections, see nature.com/documents/nr-reporting-summary-flat.pdf

Life sciences study design

All studies must disclose on these points even when the disclosure is negative.

Sample size	Sample size was determined based on our previous experimental results with the effects of rapamycin, as published in Arriola Apelo et al, 2016, Aging Cell, and the availability of DL001. For in vivo experiments, we utilized larger vehicle and rapamycin treated cohorts and smaller DL in order to reach similar statistical power (> 90% power to detect a change in area under the curve during a glucose tolerance test, $p < 0.05$) as based on the results of our previously published research.
Data exclusions	Data was not excluded. Viable splenocytes were not successfully recovered from one rapamycin-treated animal and were thus not analyzed by flow cytometry.
Replication	The positive and negative controls (vehicle and rapamycin treatments) successfully reproduced the results found in our previous studies as well as those from other laboratories. All cell culture data represents the results of at least three biologically independent experiments. All data derived from animal experiments likewise represent the results obtained from at least three biological independent animals.
Randomization	Animals were randomized into groups of equivalent weight prior to the beginning of the in vivo studies.
Blinding	Investigators were not blinded to group allocation during data collection or analysis, as cages were clearly marked to indicate the presence of a potential chemical hazard (rapamycin or DL001). This information was masked prior to the performance of in vivo GTT/PTT assays, making it less accessible to the individual investigators. Differences in group size also made blinding impractical. However, blinding is not relevant the studies conducted here, as the data is collected exclusively in numeric form which is not readily subject to bias by subjective interpretation.

Reporting for specific materials, systems and methods

We require information from authors about some types of materials, experimental systems and methods used in many studies. Here, indicate whether each material, system or method listed is relevant to your study. If you are not sure if a list item applies to your research, read the appropriate section before selecting a response.

Materials & experimental systems

n/a	Involved in the study	
☐	☒	Antibodies
☐	☒	Eukaryotic cell lines
☒	☐	Palaeontology
☐	☒	Animals and other organisms
☒	☐	Human research participants
☒	☐	Clinical data

Methods

n/a	Involved in the study	
☒	☐	ChIP-seq
☐	☒	Flow cytometry
☒	☐	MRI-based neuroimaging

Antibodies

Antibodies used	Antibodies to phospho-AKT S473 (4060), AKT (4691), phospho-p70 S6 kinase (9234), p70 S6 kinase (2708), phospho-S6 ribosomal protein (2215 or 5364), S6 ribosomal protein (2217), p-4EBP1 S65 (9451), p-4EBP1 T37/S46 (2855), total 4EBP1 (9452), p-S757 ULK1 (14202), ULK1 (8054), mTOR (2792), RICTOR (2140), HSP90 (4877), β-tubulin (2128S), and FKBP51 (12210S), and p-PKA substrate (9621) were from Cell Signaling Technology.<br>β-actin (A2228) was from Sigma, ATGL (SC-365278) was from Santa Cruz, and FKBP12 (ab2918) was from Abcam.<br>For immunoprecipitation, antibody to RICTOR (A300-458A) was from Bethyl Laboratories and Protein G Agarose (PI20398) was from Fisher.<br>For flow cytometery, antibodies to CD4 (75-0041-U025) and CD8 (80-0081-U025) were from Tonbo.<br>Antibodies to CD25 (47-0251-80) and FOXP3 (25-5773-82) were from eBioscience.<br>Antibodies to CD11a (562809553121) and CD3 (562332563204L) were from BD.
Validation	All antibodies were validated for use in mice by the manufacturers; additionally, specificity of phospho-specific mTORC1 and mTORC2 substrate antibodies has been validated by the authors and others in previous publications, including Arriola Apelo et al, 2016, Aging Cell; Lamming et al, 2014, Aging Cell; Thoreen et al, 2009, JBC. The specificity of the antibodies against FKBP12 and FKBP51 was verified in this manuscript and in Schreiber et al, 2015, Aging Cell by western blotting of cells lines in which shRNA was used to decrease expression of these proteins.

Eukaryotic cell lines

Policy information about cell lines

Cell line source(s)	All of the cell lines were obtained from ATCC except for MEFs from the Kwiatkowski lab.
Authentication	ATCC cell lines were authenticated by ATCC
Mycoplasma contamination	All cell lines in our laboratory are routinely tested for mycoplasma, and no mycoplasma contamination was detected in any cell line in the lab during the period the cell culture work was conducted.
Commonly misidentified lines (See ICLAC register)	N/A

Animals and other organisms

Policy information about studies involving animals; ARRIVE guidelines recommended for reporting animal research

Laboratory animals	C57BL/6J female and male mice at 9-10 weeks of age were used.
Wild animals	N/A
Field-collected samples	N/A
Ethics oversight	Animal studies conducted at the Buck Institute for Research on Aging were approved by the Institutional Animal Care and Use Committee (IACUC) at the Buck Institute for Research on Aging, Novato, CA. Animal studies conducted at WSM VA Hospital were approved by the IACUC of the William S. Middleton Memorial Veterans Hospital, Madison, WI.

Note that full information on the approval of the study protocol must also be provided in the manuscript.

Flow Cytometry

Plots

Confirm that:

☒ The axis labels state the marker and fluorochrome used (e.g. CD4-FITC).

☒ The axis scales are clearly visible. Include numbers along axes only for bottom left plot of group (a 'group' is an analysis of identical markers).

☒ All plots are contour plots with outliers or pseudocolor plots.

☒ A numerical value for number of cells or percentage (with statistics) is provided.

Methodology

Sample preparation	Splenocytes were prepared following a procedure from Life Technologies. Briefly, spleens were collected in a buffer containing PBS, 0.1% BSA, and 0.6% Na-citrate, and then macerated through a 70?M filter using a syringe plunger. Following centrifugation at 300 x g for 10 minutes, cells were resuspended in the same buffer, and recentrifuged. The splenocytes were suspended in PBS and 0.1% BSA with Ca2+ and Mg2+, and incubated with approximately 100 units of DNAase (Sigma). Splenocytes were then filtered through a 40?M filter and red blood cells were lysed. Splenocytes were then centrifuged, suspended in PBS with 0.1% BSA, , and brought to the UWCCC Flow Cytometry Lab for immunostaining and flow cytometry
Instrument	BD LSRII (San Jose, California)
Software	Data was collected using BD FACSDiva, Version 8.0 and analyzed with FlowJo X, Version 10.0.7r2 (FlowJo, LLC, Ashland, OR).
Cell population abundance	The abundance of cell populations is described as a percentage of live cells as identified by reaction with an amine reactive dye, Ghost510-A. Post-sort fractions were not collected for further analysis.
Gating strategy	Fluorescence minus one (FMO) controls were used to define gates for CD25, FoxP3 and LFA-1. Initial gating was performed on SSC-A and FSC-A to identify main cell population followed by gating on FSC-H and FSC-A and then SSC-H and SSC-A to identify single cells. Dead cells are excluded by an amine reactive dye, Ghost510-A. CD3+ Lymphocytes were then gated using Ghost510-A and CD3-PE-CF594-. Subsequent gating discriminated CD4+ T cells and CD8+ T Cells. Within CD8+ T cells, all cells were LFA-1+ using the FMO control to gate, so LFA-1 “Hi” cells were gated. CD25 and FoxP3 gates were set using FMO controls. Within CD4+ T cells, all cells were LFA-1+ using the FMO control to gate, so LFA-1 “Hi” cells were gated. CD25 and FoxP3 gates were set using FMO controls. LFA-1 “Hi” cells were gated within the CD4+CD25+FoxP3- gate and the CD4+CD25+FoxP3+ gate.

☒ Tick this box to confirm that a figure exemplifying the gating strategy is provided in the Supplementary Information.
